# The sucrose synthase gene family in blueberry (*Vaccinium darrowii*): functional insights into the role of *VdSUS4* in salt stress tolerance

**DOI:** 10.3389/fpls.2025.1581182

**Published:** 2025-06-02

**Authors:** Yanwen Wang, Lei Yang, Wenzhu Geng, Hongxia Zhang, Houjun Zhou

**Affiliations:** ^1^ The Engineering Research Institute of Agriculture and Forestry, Ludong University, Yantai, Shandong, China; ^2^ College of Horticulture, Ludong University, Yantai, Shandong, China; ^3^ Yantai Technology Center of Characteristic Plant Gene Editing and Germplasm Innovation, Ludong University, Yantai, Shandong, China; ^4^ Research and Development Department, Bestplant (Shandong) Stem Cell Engineering Co., Ltd., Yantai, Shandong, China; ^5^ Zhaoyuan Shenghui Agricultural Technology Development Co., Ltd, Zhaoyuan, Shandong, China

**Keywords:** blueberry, sucrose synthase, bioinformatics, tissue-specific expression, salt stress

## Abstract

**Introduction:**

The sucrose synthase (SUS), a crucial enzyme in the sucrose metabolism, is encoded by a multigene family in plant kingdom.

**Methods:**

In our study, we utilized bioinformatics tools to identify and characterize the members of the *SUS* gene family within the blueberry genome. Our analysis encompassed the physicochemical properties, gene structures, conserved motifs, promoter *cis*-acting elements, chromosomal locations, evolutionary relationships and expression profiles of these family members, allowing us to predict their potential functions.

**Results:**

We identified seven distinct *SUS* genes, mapped across six chromosomes, showcasing the complexity of this gene family in blueberries. Phylogenetic analysis, constructed through a multi-species phylogenetic tree, revealed that the *SUS* gene family can be categorized into three subfamilies: SUS I, SUS II and SUS III. Notable variations were observed among the *VdSUS* gene family members, particularly in the number of amino acids, molecular weight, isoelectric point, and hydrophobicity of the encoded proteins. Intriguingly, our predictive analysis of the promoter regions of *VdSUS* genes uncovered a wealth of cis-acting elements linked to light response, hormonal regulation, and stress responses, suggesting a role in adaptive mechanisms. Expression studies indicated that *VdSUS* genes were highly expressed in fruit tissues, with the application of exogenous sucrose leading to significant downregulation of *VdSUS2, VdSUS3* and *VdSUS6*. Furthermore, the expression of VdSUS genes was found to be responsive to abiotic stresses, such as salt, drought, and low temperatures, with varying degrees of upregulation or downregulation observed. Most notably, the overexpression of *VdSUS4* in *Arabidopsis thaliana* resulted in enhanced tolerance to salt stress.

**Discussion:**

These findings have shed new light on the multifaceted roles of *VdSUS* gene family members in the complex physiological processes of blueberries, highlighting their potential in the context of stress adaptation and fruit development.

## 1 Introduction

In the plant kingdom, sucrose serves as a vital source of carbon and energy, underpinning cellular life cycles (Huang et al., 2021). It is the predominant carbohydrate produced during photosynthesis and is subsequently transported to non-photosynthetic organs via the phloem (Liu and Zheng, 2022). Sucrose is implicated in a myriad of metabolic pathways that are central to plant growth and development. These include critical processes such as cell division, tissue differentiation, seed germination, flowering initiation, fruit maturation, and the accumulation of metabolic products. Sucrose also plays a pivotal role in responding to both biotic and abiotic stresses, as well as facilitating recovery from damage, as highlighted in various studies (Huang et al., 2021; Gaudin et al., 2000; Iraqi and Tremblay, 2001; Rook et al., 2001; Yang et al., 2001).

In higher plants, sucrose synthesis occurs via two pathways: (1) Sucrose-phosphate synthase (SPS) catalyzes the formation of sucrose-6F-phosphate (Suc6P), which is then hydrolyzed to sucrose by sucrose-phosphatase (SPP); (2) Sucrose synthase (SUS) catalyzes the conversion of UDP-glucose and fructose to sucrose and UDP, though this process is reversible (Huber and Huber, 1996; Lunn and MacRae, 2003). Research has shown that Trehalose 6-phosphate (Tre6P) functions as a specific signal for sucrose, with Tre6P levels regulating sucrose concentration. When sucrose levels increase or decrease, corresponding changes in Tre6P levels occur due to the relative activities of trehalose 6-phosphate synthases (TPS) and trehalose 6-phosphate phosphatases (TPP). In source leaves, Tre6P modulates sucrose synthesis by influencing sucrose levels, while in sink organs it regulates sucrose consumption (Figueroa and Lunn, 2016; Yadav et al., 2014). Furthermore, sucrose has been established as a signaling molecule that modulates gene expression and shapes enzyme metabolic pathways (Ciereszko et al., 2001). Sucrose specifically promotes the expression of the *Ugp* gene, which encodes UDP-glucose pyrophosphorylase. This enzyme converts UDP-glucose, produced via sucrose cleavage by SUS, into glucose-1-phosphate (Yoon et al., 2021). For instance, when detached Arabidopsis leaves are fed with 50 mM sucrose, *Ugp* gene expression is upregulated (Ciereszko et al., 2001). In isolated barley leaves, exogenous sucrose application increases fructan concentration by inducing the expression of the *fructan 6-fructosyltransferase* (*6-SFT*) gene (Nagaraj et al., 2001).

Prior to its transport to recipient tissues within the plant, sucrose is subjected to hydrolysis by two key enzymes. Invertase (INV) is responsible for breaking down sucrose into glucose and fructose, while SUS facilitates the transformation of sucrose and uridine diphosphate (UDP) into UDP-glucose and fructose (Chourey et al., 1998; Yoon et al., 2021). These enzymes are crucial for sucrose unloading in the phloem, with SUS playing a key role in carbon partitioning, biomass accumulation, and sink tissue strength (Stein and Granot, 2019). SUS has been shown to regulate the distribution of sucrose between source and sink tissues (Haigler et al., 2001), and to influence starch synthesis (Barratt et al., 2001). It also contributes to cellulose synthesis in secondary cell walls (Albrecht and Mustroph, 2003), impacts nitrogen fixation (Baier et al., 2010), and is associated with survival rates following exposure to stress (Harada et al., 2005). These functions underscore the multifaceted importance of SUS in plant physiology and adaptation.

SUS, a key player in carbohydrate metabolism, is encoded by a compact yet diverse multigene family that can be found across both monocotyledonous and dicotyledonous plants. The *SUS* gene family size differs among species: it comprises six members in *Arabidopsis* (Baud et al., 2004), rice (Hiros et al., 2008), cocoa (Li et al., 2015), tomato (Duan et al., 2021), and citrus (Islam et al., 2014), five in grape (Zhu et al., 2017), and three in maize (Duncan et al., 2006). Notably, cotton has seven SUS genes (Chen et al., 2012), apple boasts eleven (Tong et al., 2018), and poplar exhibits an expansive family of fourteen (An et al., 2014). Remarkably, the largest *SUS* gene family, with an impressive thirty members, has been identified in pear (Harada et al., 2005). Variation is a hallmark of the *SUS* gene family across species, with each member exhibiting unique functional roles and structural characteristics. Their expression patterns also diverge, highlighting distinct stages of plant growth and development. For example, in pea, the *SUS* gene family is divided into three clear subfamilies SUS I, SUS II, and SUS III each with its own distinct expression profile during organogenesis. SUS I members demonstrate broad tissue expression and are particularly abundant in developing seeds. In contrast, SUS II members are predominantly expressed in mature ovaries and leaves, while SUS III members exhibit limited expression, primarily in flowers and young ovaries (Barratt et al., 2001). This tissue-specific expression pattern underscores the evolution of *SUS* genes towards specialized physiological functions, reflecting their intricate involvement in the developmental and adaptive processes of plants.

In maize, *SUS* genes associated with cell wall synthesis are abundantly expressed in developing endosperm, while *ZmSUS1* is widely expressed and plays a central role in starch synthesis (Duncan et al., 2006). Moreover, the overexpression of *ZmSUS1* significantly improves maize seed traits, increasing starch content (Li et al., 2023). In *Arabidopsis*, the six *AtSUS* genes also exhibit differential expression, and extensive research has been conducted on their specific functions in studies involving knockout mutants (Baud et al., 2004; Bieniawska et al., 2007). Mutants of pea *SUS* (*rug4*) exhibit reduced seed starch content, while the overexpression of *SUS* in potato stems leads to starch accumulation (Barratt et al., 2001; Zrenner et al., 1995; Baroja-Fernández et al., 2009). The wheat *SUS* gene *TaSUS1* is a determinant of grain number per spike (Shen et al., 2023). In hybrid aspen *(Populus tremula* × *tremuloides*), specific reduction of *SUS* (*PtrSUS1* and *PtrSUS2*) expression levels in wood through *RNAi* technology, leads to changes in cell wall structure and significant reduction in wood density in the transgenic lines (Gerber et al., 2014). Similarly, overexpression of *PsnSUSy1* and *PsnSUSy2* genes in tobacco thickens the secondary cell wall, enhancing nutritional growth and mechanical strength (Li et al., 2019). In stress studies, the *Arabidopsis sus1*/*sus4* double mutant exhibits normal growth but shows significant growth retardation when the roots are subjected to hypoxic conditions (Bieniawska et al., 2007). In cucumber, the expression and activity of *CsSUS3* increase when subjected to flooding low-oxygen stress, especially in lateral roots (Wang et al., 2014).

Blueberry, recognized as one of the five major health foods for humans and hailed as the “king of fruits worldwide”, is known for its delicious taste and rich content of functional components such as organic acids, phenolics, minerals and vitamins. It possesses medicinal value with antioxidant, anti-inflammatory, anti-cancer, neuroprotective and vision-improving properties (Duan et al., 2022). The storage of sugars in blueberry fruits begins with SUS, which converts fructose and UDP-glucose into sucrose and UDP. Understanding the function of SUS in sucrose synthesis and cleavage is crucial for addressing fruit over-acidification and enhancing the quality of blueberry fruits.

In this study, using blueberry (O’ Neal) as the material, we identified members of the *SUS* gene family from the genome database of evergreen blueberry (*Vaccinium darrowii*). We conducted bioinformatic analysis, including chromosome localization, gene structure, conserved motifs and evolutionary relationships. Additionally, we investigated the expression patterns of *VdSUS* genes in different tissues and developmental stages. By externally applying sucrose to blueberry fruits, we analyzed the role of *VdSUS* genes in fruit ripening. We also investigated the response of the *VdSUS* genes to abiotic stress. Moreover, overexpression of the *VdSUS4* gene enhanced salt tolerance in transgenic *Arabidopsis*. In summary, our results contributed to a comprehensive understanding of physiological functions of the blueberry *SUS* gene family in abiotic stress tolerance.

## 2 Materials and methods

### 2.1 Identification of *VdSUS* gene family members in blueberry

We obtained the blueberry protein sequences from the NCBI database (https://www.ncbi.nlm.nih.gov/). The SUS family protein domain feature files (PF00534 and PF00862) were obtained from the Pfam website (https://pfam.xfam.org/). A Hidden Markov Model (HMM) was built using HMMER 3.0, and the hmmsearch program from HMMER 3.0 was employed to search for protein sequences containing the SUS family protein domain features in the blueberry protein database. Redundant protein sequences were manually removed. A total of 7 protein sequences were identified as candidate members of the blueberry SUS family. The identified protein sequences were validated for conserved domains using NCBI-CDD (https://www.ncbi.nlm.nih.gov/cdd/) and SMART (http://smart.embl-heidelberg.de/). The physicochemical properties of the *VdSUS* gene family proteins, including the number of amino acids, molecular weight, theoretical isoelectric point and hydropathicity, were predicted using the ExPASy (https://web.expasy.org/protparam/) online website. The subcellular localization of VdSUS proteins were predicted using the website (http://www.genscript.com/tools/wolf-psort).

### 2.2 Chromosome localization, sequence alignment, gene structure, conserved motifs and three-dimensional structural domain analysis

According to information from the GFF annotation file (NCBI), the TBtools software was employed to visualize the chromosome positions and gene structures of *VdSUS* gene family (Chen et al., 2020). Sequence alignment of SUS protein sequences was conducted using Jalview software. The analysis of conserved motifs was performed using the online tool MEME (https://meme-suite.org/meme/). Three-dimensional structural analysis of VdSUS protein sequences was carried out using the online tool SWISS-MODEL (https://swissmodel.expasy.org/). To ensure the accuracy of the model, AtSUS1, which shares high sequence similarity with VdSUS, was used as a template.

### 2.3 Phylogenetic tree analysis of the *SUS* gene family across multiple species

To construct a systematic phylogenetic tree of SUS, protein sequences of SUS from *Arabidopsis*, maize, rice, tomato, wheat, and sugar beet were extracted from previous studies. Multiple sequence alignment was performed using MEGA X software, and a neighbor-joining (NJ) method was employed to build the phylogenetic tree with a bootstrap value of 1000 (Kumar et al., 2018). Subsequently, the tree was visualized and enhanced using the online tool Evolgenius (https://evolgenius.info//evolview-v2).

### 2.4 Collinearity analysis

Downloaded the genome files and GFF files for *Arabidopsis*, rice, and grape from the NCBI database. Utilized the multiple collinearity scanning toolkit in MCScanX within TBtools for the analysis of cross-species collinearity relationships, followed by visualization using TBtools (Chen et al., 2020).

### 2.5 The analysis of the promoter cis-elements of *VdSUS*

A 2 kb DNA sequence upstream of the start codon (ATG) was extracted from the *VdSUS* gene family. Predictions for *cis*-acting elements were performed using the Plant-CARE (http://bioinformatics.psb.ugent.be/webtools/plantcare/html/) website (Higo et al., 1999). Subsequently, a classification analysis was conducted using Excel, and visualization was performed using TBtools.

### 2.6 Plant materials, growth conditions, sucrose and stress treatments

This study utilized blueberry (O’Neal) and *Arabidopsis thaliana* (Columbia-0) as experimental materials. Blueberry and *Arabidopsis* plants were cultivated in plant growth chambers under 16 h of light and 8 h of darkness, with a light intensity of 100 μmolm^−2^s^−1^, and temperatures at 23°C (light) and 20°C (dark). Various tissues, representing different developmental stages and tissue types, were collected from soil-grown blueberry plants, including roots, young stems, mature stems, young leaves, mature leaves, young flowers, mature flowers, early green fruits, late green fruits and mature fruits.

Sucrose treatment involved the external application of sucrose to late green fruits. Uniform-sized fruits were selected, and 100 μL 50 mM sucrose was injected into the fruits using 1 mL syringe. And 100 μL 50 mM sorbitol served as an osmotic potential control. Samples were collected at 0 h, 6 h, 12 h and 24 h post-treatment. Additionally, blueberry seedlings were subjected to drought, salt and low-temperature (4°C) treatments. For drought treatment, seedlings were exposed to drought for 0–15 d. Salt stress was treated using 200 mM NaCl for 0–11 d. Low-temperature stress involved placing the seedlings in a 4°C incubator for 0–24 h after pre-cooling the incubator to 4°C the day before. After treatment, plant leaves were immediately frozen in liquid nitrogen and stored at -80°C for further analysis.

For salt stress treatment, wild-type and transgenic *Arabidopsis* seeds were surface-sterilized and sown on Murashige and Skoog medium (pH 5.9) supplemented with 100 mM NaCl. The plates were placed at 4°C for 2 days, and then at 22°C for vertical growth. Phenotypic images were captured, and root length and fresh weight were recorded. In addition, wild-type and transgenic Arabidopsis seeds were surface-sterilized and sown on 1/2 MS medium. After 7 days of cultivation, seedlings were transplanted into pots (10 × 10 cm) filled with a 1:1 mixture of nutrient soil and vermiculite, and then cultured in a plant growth chamber. Three weeks post-transplantation, healthy wild-type and transgenic Arabidopsis plants were irrigated with equivalent volumes of 150 mM NaCl solution. Growth performance was subsequently monitored and recorded. Regarding the measurement of seed germination rate, surface-sterilized seeds were sown on 1/2 MS medium, and germination rates were assessed after 4 d of incubation based on the emergence of radicles and cotyledons.

### 2.7 Physiological analysis

Following salt stress treatment, collected leaf samples (0.2 g) were homogenized in 5 mL of ice-cold 25 mM phosphate-buffered saline (PBS, pH 7.8) containing 0.2 mM EDTA. POD (peroxidase), SOD (superoxide dismutase), CAT (Catalase) activities and MDA contents were determined using commercial assay kits according to the manufacturer’s instructions (Nanjing Jiancheng Bioengineering Institute, China).

### 2.8 Vector construction and plant transformation

To construct the plant expression vector pCAMBIA1300-*VdSUS4* under the control of the Cauliflower Mosaic Virus (CaMV) 35S promoter, the complete coding sequence of *VdSUS4* was inserted into the pCAMBIA1300 vector using the ClonExpress II One Step Cloning Kit (Vazyme, China). The recombinant vector was transformed into *Agrobacterium tumefaciens* strain GV3101, and the transformation of *Arabidopsis* plants was performed as previously described (Clough and Bent, 1998). Homozygous transgenic plants were selected on 1/2 MS medium containing 50 mg/L kanamycin for subsequent experiments.

### 2.9 RNA extraction and quantitative real-time PCR analysis

Total RNA extraction was performed using the FastPure Universal Plant Total RNA Isolation Kit (Vazyme, China), and cDNA synthesis was conducted using the HiScript III 1st Strand cDNA Synthesis Kit (Vazyme, China). The reference gene *VdTub2* (*Vda09G008900.1*) was utilized, and quantitative PCR was carried out using the CFX Connect Real-Time System (Bio-Rad, America) with ChamQ Universal SYBR qPCR Master Mix (Vazyme, China). Gene-specific primers for *VdSUS* were designed using Premier 5.0 software, and the sequences are listed in **Supplementary Table S3**. The reaction conditions were as follows: pre-denaturation at 95°C for 1 min, denaturation at 95°C for 15 s, annealing/extension at 60°C for 15 s, and a total of 40 cycles. The comparative CT method was employed to assess the relative expression levels of qRT-PCR products (Schmittgen and Livak, 2008).

### 2.10 Data analysis

Data were organized and categorized using Excel (Version 2019), visualized with TBtools (Version 2.042), and refined in Adobe Illustrator 2020. Statistical analyses were carried out using Prism 8 and SPSS 20. All results are presented as means ± standard deviation (SD) from three biological replicates. Significant differences were analyzed by Student’s t-test.

## 3 Results

### 3.1 Identification and characterization of the blueberry *SUS* gene family members

To elucidate the biological functions of the *SUS* gene family in blueberries, we constructed a Hidden Markov Model. Seven protein sequences containing the Sucrose_synth and Glyco_trans_1_4 domains were identified in the blueberry whole genome. Based on their phylogenetic relationship with *Arabidopsis*, they were named *VdSUS1*-*VdSUS7* (**Supplementary Figure S1**). The amino acid sequences of the blueberry *SUS* gene family ranged from 811 to 1059. The molecular weights ranged from 92254.59 (VdSUS3) to 119144.5 (VdSUS7), with isoelectric points between 5.73 (VdSUS3) and 8.16 (VdSUS5). The instability index ranged from 28.97 (VdSUS6) to 92.68 (VdSUS2), and all had negative average hydrophobicity coefficients, indicating hydrophilic proteins. Subcellular localization prediction indicates that VdSUS may be located in the cytoplasm, nucleus, and mitochondria (**Table 1**).

**Table 1:** The main detail characteristics of 7 VdSUSs proteins in blueberry.

Gene ID		DNA attributes	Protein attributes					
Gene name	Gene locus (MSU)	Nucleotide length (bp)	Instability index	GRAVY	Length (aa)	MW (Da)	PI	Subcellular localization
*VdSUS1*	Vadar_g22938	2418	-0.252	92.51	806	92733.84	6.16	Cyto,Chlo
*VdSUS2*	Vadar_g38054	2433	-0.253	92.68	811	92430.52	5.81	Cyto
*VdSUS3*	Vadar_g7208	2433	-0.247	92.33	811	92254.59	5.73	Mito, Chlo, Cyto
*VdSUS4*	Vadar_g10639	3159	-0.456	46.92	1,053	118496.52	6.11	Nucl,Chlo,Cyto
*VdSUS5*	Vadar_g41615	2700	-0.337	42.41	900	101924.11	8.16	Cyto,Nucl,Mito
*VdSUS6*	Vadar_g25886	2454	-0.324	28.97	818	92467.95	7.87	Cyto
*VdSUS7*	Vadar_g37562	3177	-0.442	85.88	1,059	119144.5	6.31	Nucl,Cyto

MW, molecular weight; PI, isoelectric point.

The conserved domains and phylogenetic relationships of VdSUS proteins were explored through a multiple sequence alignment of the *VdSUS* gene family. The results of the multiple sequence alignment showed that all seven VdSUS proteins possessed two domains, Sucrose_synth and Glyco_trans_1_4 (**Supplementary Figure S2A**). Analysis of the position of these two domains in the protein sequences revealed that the domain sequences of VdSUS4 and VdSUS7 were slightly shorter than those of the other members (**Supplementary Figure S2B**). The amino acid sequences of the *SUS* gene family proteins exhibited a predominance of *α*-helix secondary structures, each exceeding 50%, with the least proportion being *β*-turn, which was around 6% (**Supplementary Figure S2C**). The tertiary structure of VdSUS proteins consisted of two symmetric tetramers (**Supplementary Figure S2D**, **Supplementary Figure S3**), forming a three-lobed structure, with four distinct domains (**Supplementary Figure S2E**). The first two domains were designated as the Cellulose Targeting Domain (CTD, residues 1–121) and the Early Nodulin 40 Peptide Binding Domain (EPBD, residues 161–271). The last two domains included the GT-B glycosyltransferase with its Rossmann fold domain (**Supplementary Figure S2E**). These folds form an active site suited for substrate binding, responsible for recognizing and binding sucrose and UDP, and participating in the catalytic reaction of glycosyl transfer. The N-terminal and C-terminal domains of GT-B glycosyltransferase are referred to as GT-B_N_ and GT-B_C_, respectively (Zheng et al., 2011). The GT-B_N_ domain extended from residues 275 to 528, and the GT-B_C_ domain extended from residues 529 to 760.

**Figure 1:**
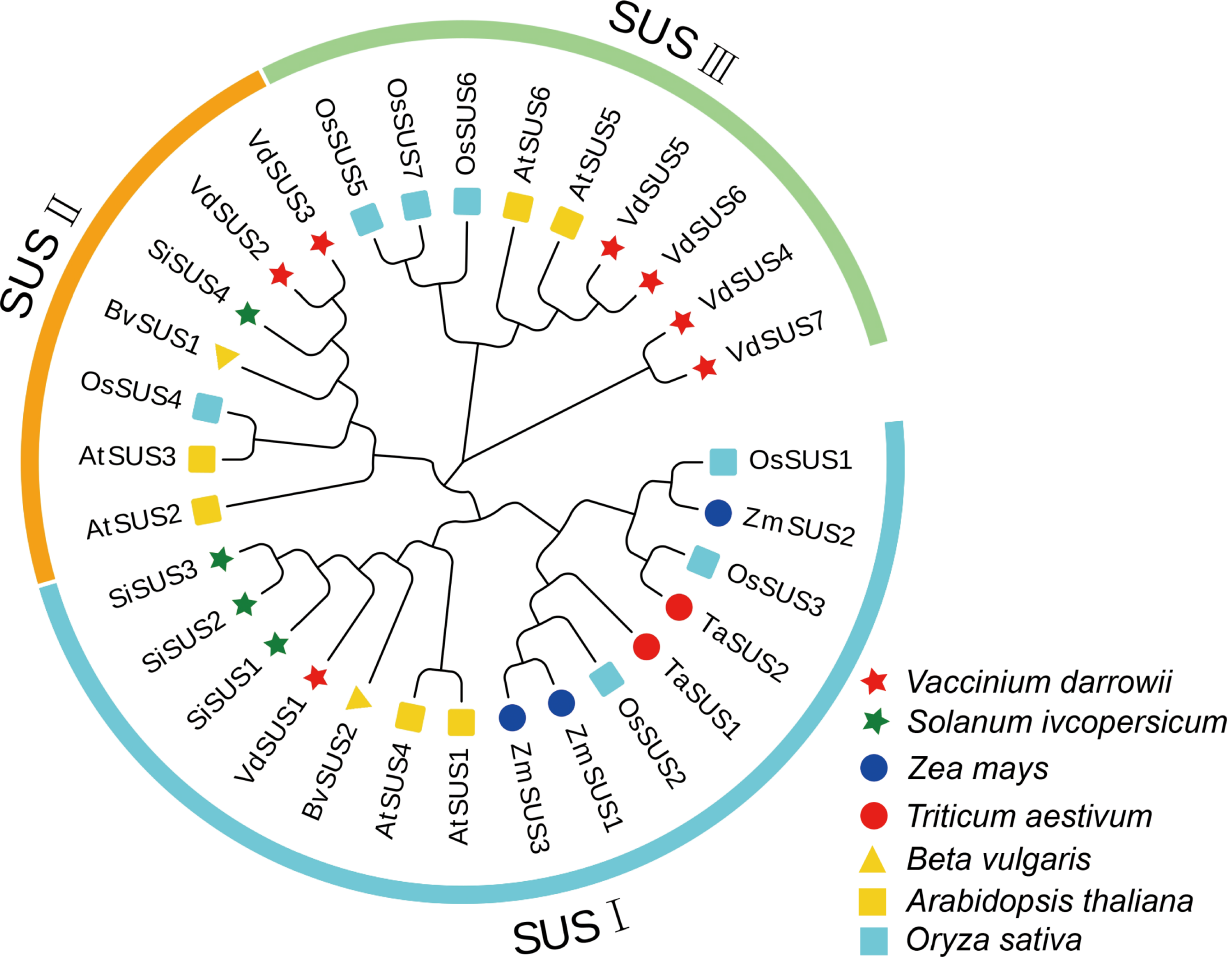
Phylogenetic analysis of the *SUS* gene family. VdSUSs were labeled with red stars.

To investigate the evolution of the *SUS* gene family across different plant species, we collated the amino acid sequences of seven VdSUS proteins from blueberry, six AtSUS proteins from *Arabidopsis* (Bieniawska et al., 2007), seven OsSUS proteins from rice, four SiSUS proteins from tomato, three ZmSUS proteins from maize, two TaSUS proteins from wheat, and two BvSUS proteins from sugar beet. We constructed a neighbor-joining (NJ) phylogenetic tree (**Figure 1**; **Supplementary Table S1**) to analyze the evolutionary relationships. The results of the phylogenetic tree analysis revealed that the 31 SUS proteins from the seven species could be divided into three groups: SUS I, SUS II and SUS III. Blueberry VdSUS proteins were distributed in each subgroup, with only one protein (VdSUS1) in the SUS I group. Notably, these three groups were present in both monocotyledonous and dicotyledonous plants, suggesting a common ancestor. Additionally, VdSUS exhibited a unique clustering pattern, but most collinear orthologous gene pairs were distributed in AtSUS and OsSUS, indicating a shared evolutionary history among these genes across different species (**Figure 1**). This observation may be attributed to evolutionary variations between monocotyledonous and dicotyledonous plants, influencing the distribution of these gene pairs in their respective species.

### 3.2 Chromosomal localization and collinearity of the blueberry *SUS* gene family

**Figure 2:**
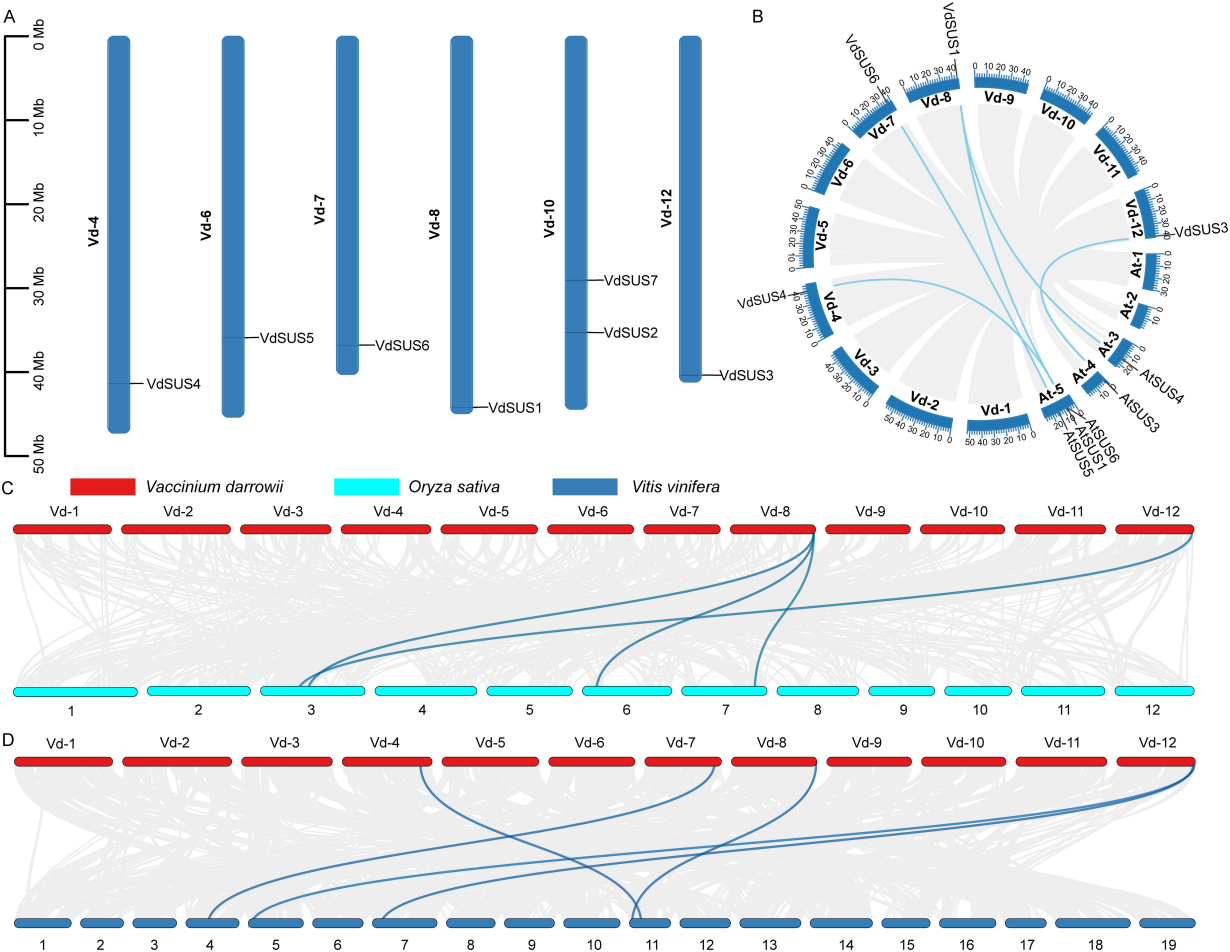
Characteristics of VdSUS proteins. **(A)** Chromosomal localization. **(B)** Collinearity relationship between blueberry and *Arabidopsis SUS* genes. **(C)** Collinearity relationship between blueberry and rice *SUS* genes. **(D)** Collinearity relationship between blueberry and grape.

To investigate the chromosomal localization of *SUS* gene in the blueberry genome, we downloaded the blueberry GFF file from NCBI and visualized it using TBtools. The results showed that the seven *SUS* genes were distributed on 6 chromosomes of blueberry (**Figure 2A**). The vd-10 chromosome has 2 *SUS* genes, while the other chromosomes have one gene each (**Figure 2A**).

Gene duplication was a common occurrence in plant evolution, including whole-genome duplication, tandem duplication and segmental duplication, which could generate homologous genes with similar sequences. We performed intraspecific collinearity analysis of the *VdSUS* gene family and found no collinear gene pairs (**Figure 2B**). To further investigate the phylogenetic and evolutionary relationships of *SUS* genes between species, and to reveal the collinearity relationships of *SUS* genes in different species, we selected three representative model species, including two dicotyledonous plants (*Arabidopsis* and grape) and one monocotyledonous plant (rice), for collinearity analysis with blueberry. These results showed that there were 5 pairs of collinear *SUS* gene pairs between blueberry and *Arabidopsis*, including *VdSUS4* and *AtSUS6*, *VdSUS6* and *AtSUS5*, *VdSUS1* and *AtSUS3*, *VdSUS1* and *AtSUS1*, and *VdSUS3* and *AtSUS3* (**Figure 2B**). In comparison with rice genome (7 genes), four collinear gene pairs were identified between *VdSUS* and *OsSUS* (**Figure 2C**; **Supplementary Table S2**). The *VdSUS* genes exhibited higher homology with the *VvSUS* genes of grape (5 genes) (**Figure 2D**; **Supplementary Table S2**), indicating a close relationship between them. These results also suggested that, compared to monocotyledonous plant genomes, blueberry exhibited more significant collinearity with dicotyledonous plant genomes, and individual homologous genes showed one-to-many or many-to-one homology. These genes had undergone multiple genes duplication events, indicating a close phylogenetic relationship between the studied species. Their evolutionary functions might be conserved, and their ancestral functions had not been lost or altered during the duplication process, playing an important role in the evolution of the *SUS* gene family.

### 3.3 Blueberry *SUS* gene family structure and conserved motif analysis

**Figure 3:**
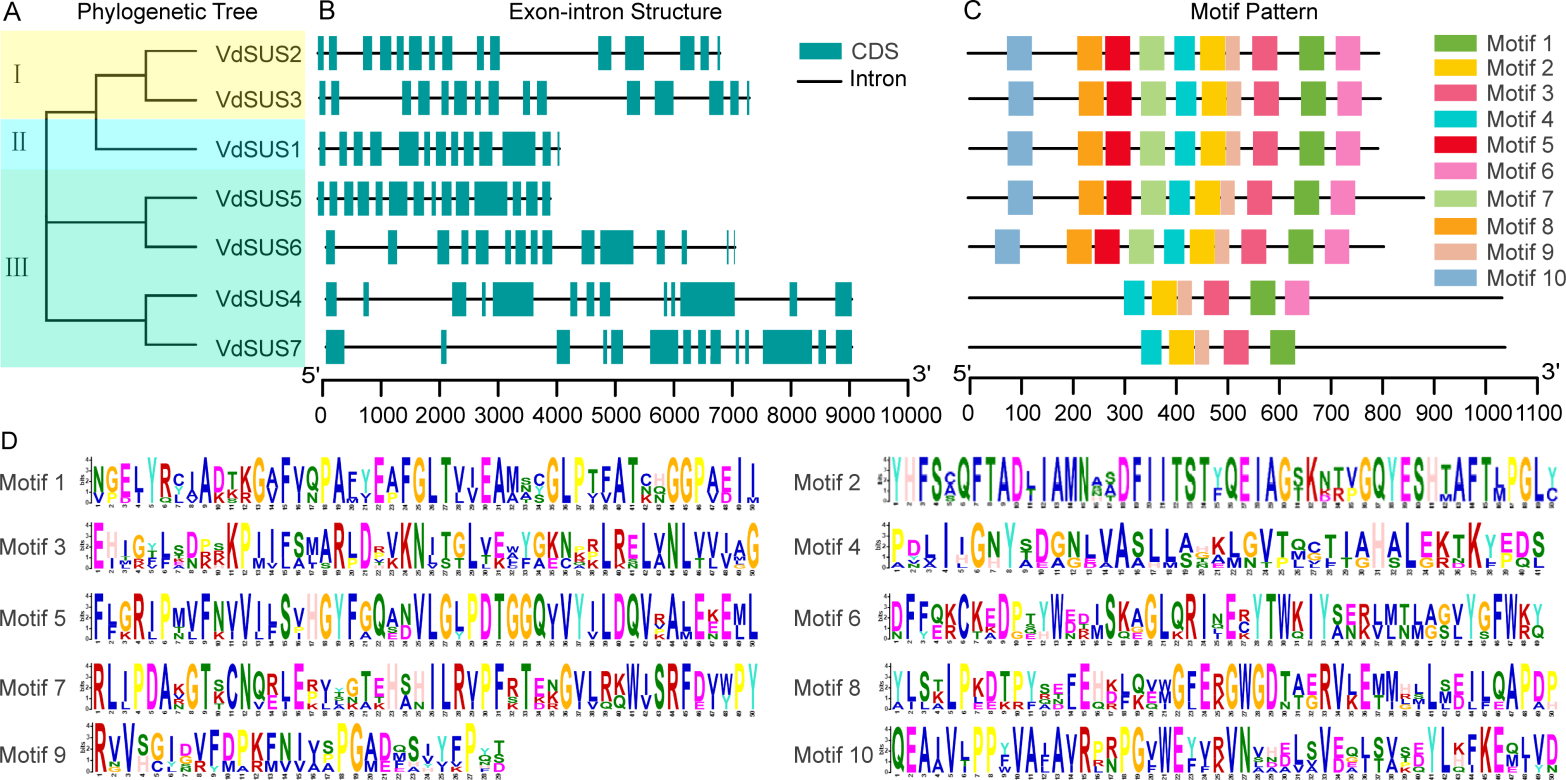
Structural features of *VdSUS* sequences. **(A)** Phylogenetic tree of the VdSUS protein family, where I represents SUS I, II represents SUS II, and III represents SUS III. **(B)** Gene structure features of the *VdSUS* genes family. **(C)** Motif analysis of the VdSUS proteins. **(D)** Conserved motif sequences.

To further investigate the structural features and evolutionary mechanisms of the *SUS* gene family, we conducted phylogenetic tree analysis of the SUS proteins in the blueberry genome, as well as a comparative analysis of the distribution of conserved motifs and intron-exon structures. Predicted gene structures revealed that *SUS* gene sequences contained 12 or more introns (**Figure 3B**). The sequences of all VdSUS proteins contained Motif 1, Motif 2, Motif 4, Motif 5 and Motif 9 (**Figure 3C**), with most motifs consisting of 50 amino acids (except for Motif 4 and Motif 9) (**Figure 3D**). VdSUS5 and VdSUS6 belonged to the SUS III group (**Figure 3A**), but they exhibited differences in gene structure while sharing the same motif distribution. This divergence in gene structure may contribute to functional differences among *VdSUS* genes. In contrast, the gene structures and conserved motifs of *VdSUS2/VdSUS3* and *VdSUS4/VdSUS7* were consistent, indicating a strong correlation between phylogenetic relationships among gene family members and their gene structures. This might suggest functional redundancy among these genes in blueberries.

### 3.4 Analysis of promoter cis-acting elements in the blueberry *SUS* gene family

**Figure 4:**
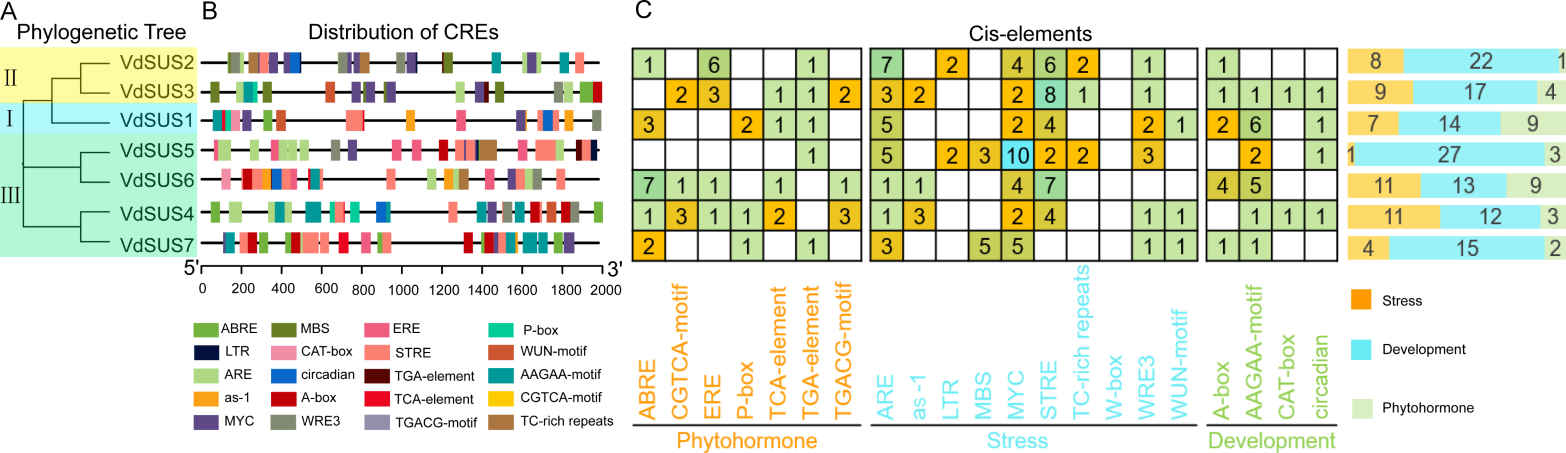
Analysis of *cis*-regulatory elements in the promoter regions of *SUS* genes in blueberry. **(A)** Phylogenetic tree of the VdSUS protein family in blueberry. **(B)** The positions of *cis*-regulatory elements in the promoter regions of *VdSUS* genes in blueberry. **(C)** Statistical analysis of the number of *cis*-regulatory elements in the promoter regions of *VdSUS* genes in blueberry.

*Cis*-regulatory elements (CREs) are non-coding DNA sequences in the gene promoter region that play a crucial role in gene expression, widely participating in the regulation of plant growth, development and stress responses (Zhao et al., 2018). By analyzing the CREs of *VdSUS*, we aimed to further understand the potential roles of *VdSUS* in plant growth and development, as well as in response to plant hormones and abiotic stress. To identify genes functions and regulatory patterns, we investigated the CREs in the promoter regions of each gene by searching the 2000 bp region around each transcription activation site in the Plant CARE database. The analysis of *VdSUS* promoter regions revealed various CREs. We selected 20 representative CREs for visualization of their distribution (**Figure 4B**). Among them, *VdSUS3* had the highest number of CREs, totaling 33, while *VdSUS7* had 21 CREs. All CREs could be broadly classified into three categories (**Figure 4C**). The first category was hormone response, with a total of 51 CREs in the *VdSUS* gene family. Some genes, such as *VdSUS4* and *VdSUS6*, contained multiple hormone response elements in their promoter regions, suggesting a rapid and intense response to specific hormones. The second category was abiotic stress, with a total of 120 CREs in the *VdSUS* gene family. All *VdSUS* genes contained abiotic stress-related elements such as ARE (anaerobic stress-related) and MYC (salt stress-related), indicating their potential role in regulating anaerobic stress and salt stress responses. The third category was growth and development, with a total of 31 CREs related to growth and development. Among them, *VdSUS1* and *VdSUS6* contained the highest number of AAGAA-motif (auxin response element), suggesting their involvement in blueberry growth and development regulation.

In summary, these results indicated that the composition and quantity of CREs in different *VdSUS* promoter regions vary among subfamilies (**Figure 4A**). This suggested that the functional expression of *SUS* genes in blueberries was regulated by various CREs associated with hormones, abiotic stress, and plant growth and development processes.

### 3.5 Expression analysis of blueberry *SUS* genes in different organs, tissues and developmental stages

**Figure 5:**
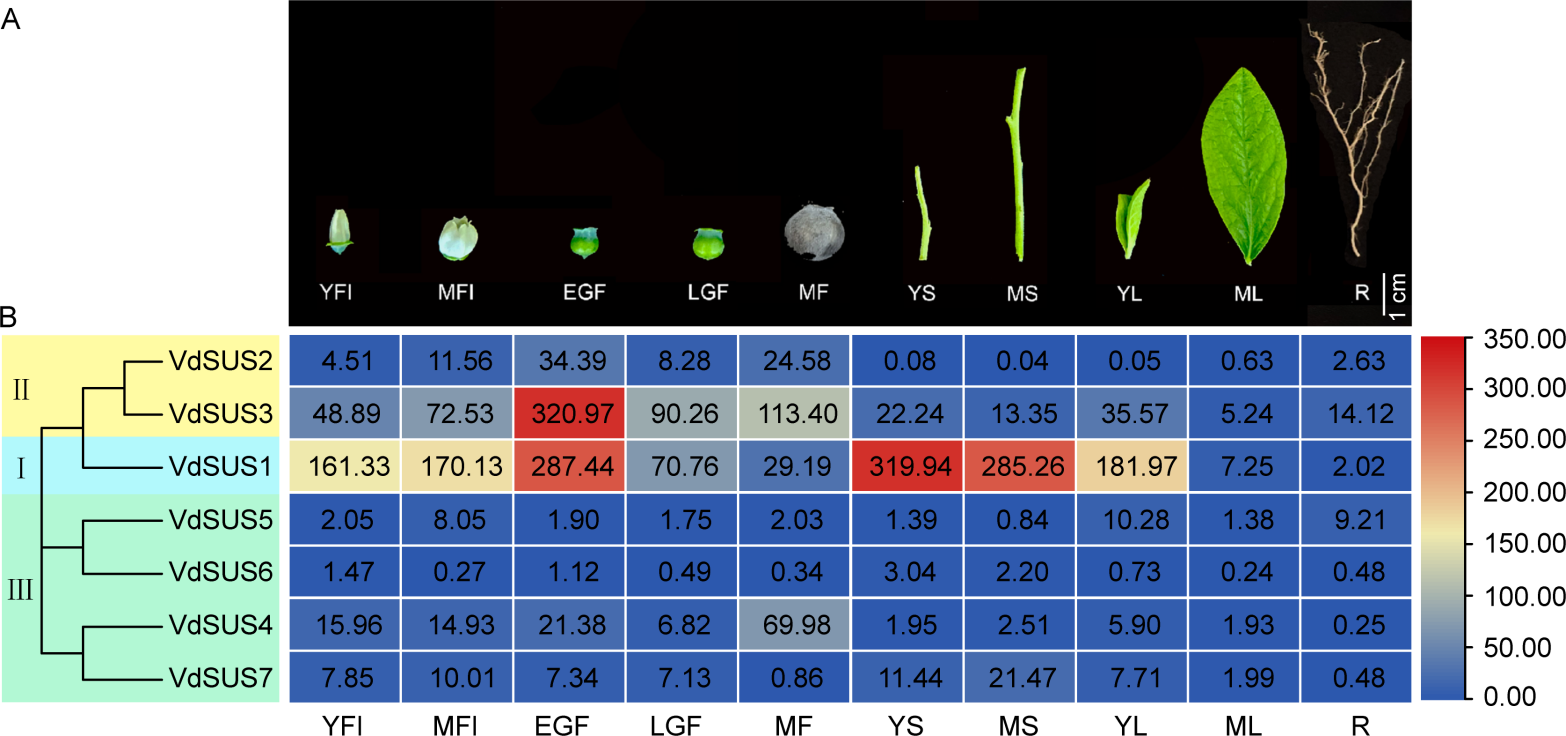
Spatial expression patterns analysis of *VdSUS* genes. **(A)** Phenotypes of extracted blueberry samples in different organs, tissues and developmental stages. YFl, young flowers; MFl, mature flowers; EGF, early green fruits; LGF, late green fruits; MF, mature fruits; YS, young stems; MS, mature stems; YL, young leaves; ML, mature leaves; R, roots. **(B)** Expression levels of *VdSUS* family genes.

To better elucidate the physiological functions of *VdSUS* gene family members, we employed qRT-PCR to examine the spatiotemporal gene expression patterns of *VdSUS* genes in different organs, tissues and developmental stages of blueberry. Fluorescence quantitative PCR primers were designed based on the CDS region of the 7 members of the *VdSUS* gene family, *VdTub2* serving as the internal reference control. The expression levels of the 7 *VdSUS* genes were detected by qRT-PCR in various tissues, including young flowers (YFl), mature flowers (MFl), early green fruits (EGF), late green fruits (LGF), mature fruits (MF), young stems (YS), mature stems (MS), young leaves (YL), mature leaves (ML) and roots (R) (**Figure 5A**). In flowers, the expression levels of most *VdSUS* genes were higher in mature flowers than young flowers. In fruits, except for *VdSUS4*, which showed higher expression in mature fruits than in early and late green fruits, other *VdSUS* members exhibited the highest expression in early green fruits, followed by a decline in late green fruits (**Figure 5B**). Interestingly, their expression levels increased again in mature fruits. In stems, the expression levels of *VdSUS1*, *VdSUS3* and *VdSUS7* were relatively high, while the expression levels of other members were generally low (**Figure 5B**). In leaves, except for *VdSUS2*, the expression levels of other members were higher in young leaves than in mature leaves (**Figure 5B**). In roots, the expression levels of *VdSUS3*, and *VdSUS5* were relatively high, while expression levels of other members were low (**Figure 5B**). Overall, *VdSUS5* and *VdSUS6* showed lower expression levels in various tissues, indicating their contribution to the growth, sucrose metabolism and fruit development in blueberries (**Figure 5B**).

### 3.6 Expression patterns of *VdSUS* genes in blueberry fruits under sucrose treatment and abiotic stress conditions

Exogenous sucrose had been demonstrated to act as a signaling molecule, promoting the ripening of tomato and strawberry fruits (Jia et al., 2013, 2016). In order to further identify which *VdSUS* genes influence the ripening and softening of blueberry fruits, the expression of *VdSUS* genes in blueberry fruits was modulated by exogenous sucrose application. The results revealed that, compared to blueberry fruits treated with sorbitol (negative control), the expression of *VdSUS4* in blueberry fruits was upregulated after 6 hours of sucrose treatment, reaching its peak after 12 h with a threefold increase compared to the negative control. *VdSUS1* and *VdSUS7* showed an upregulation in expression after 24 h of sucrose treatment, while expression of *VdSUS2*, *VdSUS3*, and *VdSUS6* exhibited a slight decrease after sucrose treatment. *VdSUS5* showed no significant change compared to the negative control (**Supplementary Figure S4**).

**Figure 6:**
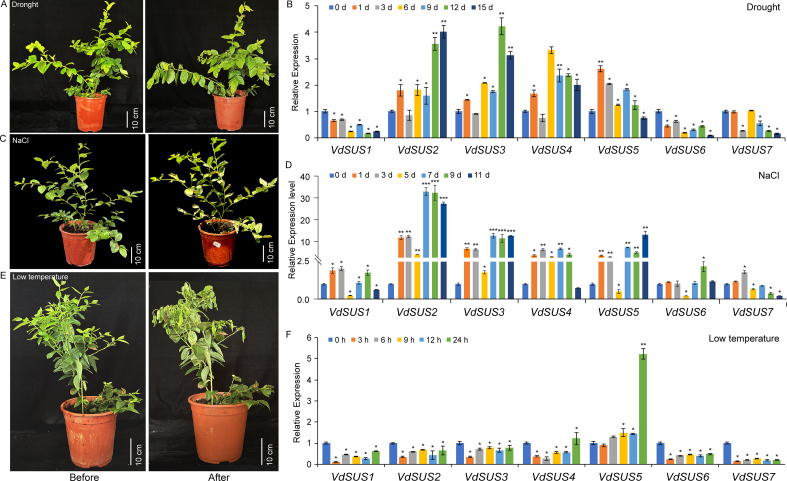
Expression patterns of *VdSUS* genes in response to abiotic stress treatment. **(A)** Phenotypic charts before and after drought treatment. **(B)** Expression levels of *VdSUS* genes under drought treatment conditions. **(C)** Phenotypic charts before and after NaCl treatment. **(D)** Expression levels of *VdSUS* genes under NaCl treatment conditions. **(E)** Phenotypic charts before and after low temperature treatment. **(F)** Expression levels of *VdSUS* genes under low temperature treatment conditions. Values are the average ± standard deviation of three biological replicates. The transcription levels of *VdSUS* genes at 0 days and 0 hours were set as “1”. *P*-values < 0.05, 0.01 and 0.001 are denoted by “*”, “**” and “***” respectively (Student’s t-test).

In order to further explore the potential role of *VdSUS* in abiotic stress, we investigated the expression patterns of *VdSUS* under drought, salt and low temperature stress conditions. The results revealed differential expression patterns of *VdSUS* under drought (**Figure 6A**), salt (**Figure 6C**), and low temperature stress (**Figure 6E**). The expression levels of *VdSUS1*, *VdSUS6* and *VdSUS7* were downregulated, while the expression levels of *VdSUS2*-*VdSUS5* were upregulated after drought treatment (**Figure 6B**). After NaCl treatment, the general trend of *VdSUS1-VdSUS5* gene expression was upregulated, and *VdSUS7* showed initial upregulation followed by downregulation at 5 d, while *VdSUS6* was almost unaffected (**Figure 6D**). Under low temperature treatment, except for *VdSUS5* was induced upregulation, all other genes were significantly downregulated (**Figure 6F**).

### 3.7 Overexpression of *VdSUS4* confers salt stress tolerance

**Figure 7:**
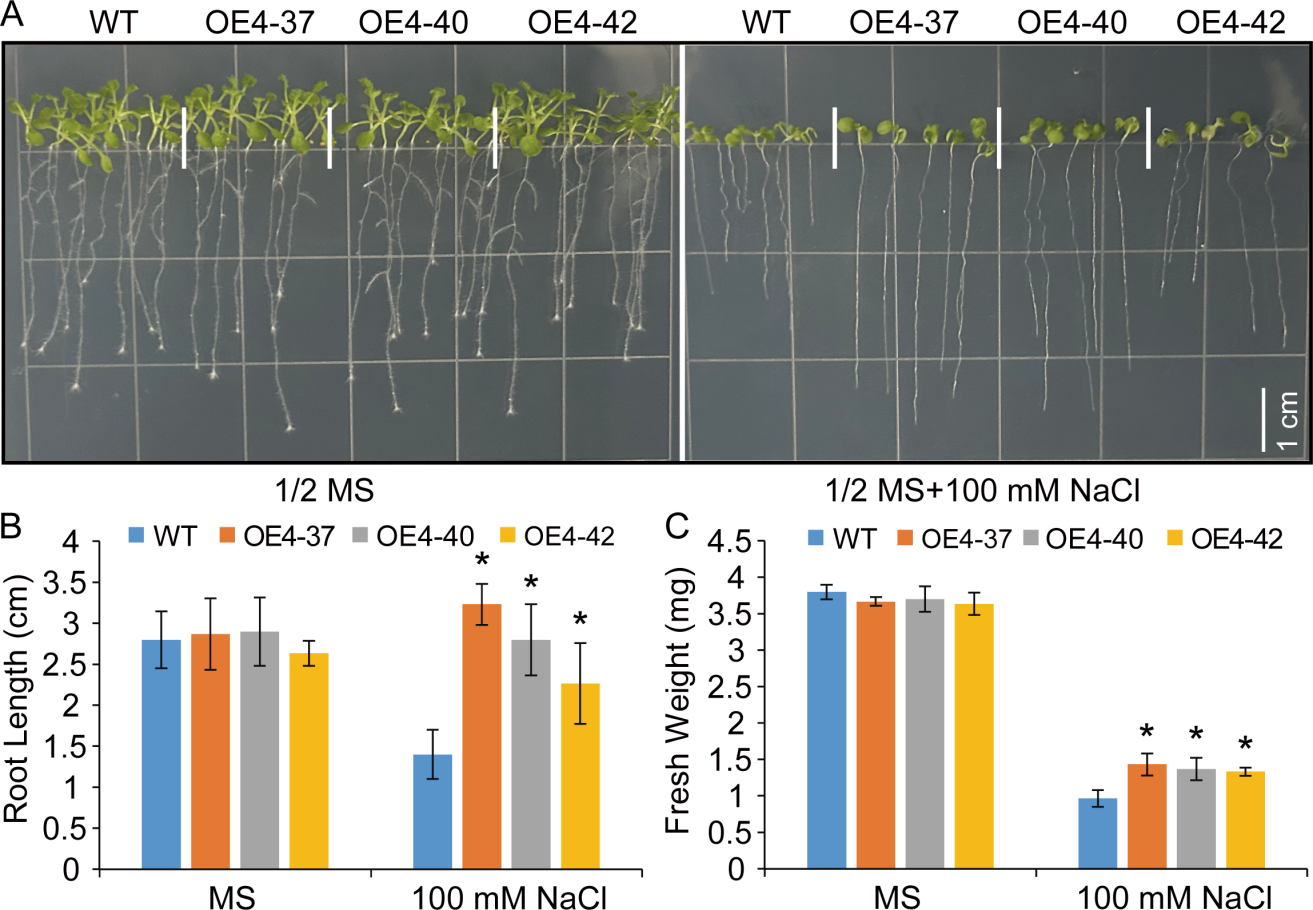
Overexpression of *VdSUS4* enhanced salt stress tolerance in *Arabidopsis*. **(A)** Germination of WT and *VdSUS4* overexpressing lines on media with or without 100 mM NaCl, photographed after 15 d. **(B)** Root length measurement of 15-day-old WT and *VdSUS4* overexpressing lines. **(C)** Fresh weight measurement of 15-day-old WT and *VdSUS4* overexpressing lines. Values are the mean ± standard deviation of three biological replicates. *p*-values < 0.05 are represented by “*” (Student’s t-test).

**Figure 8:**
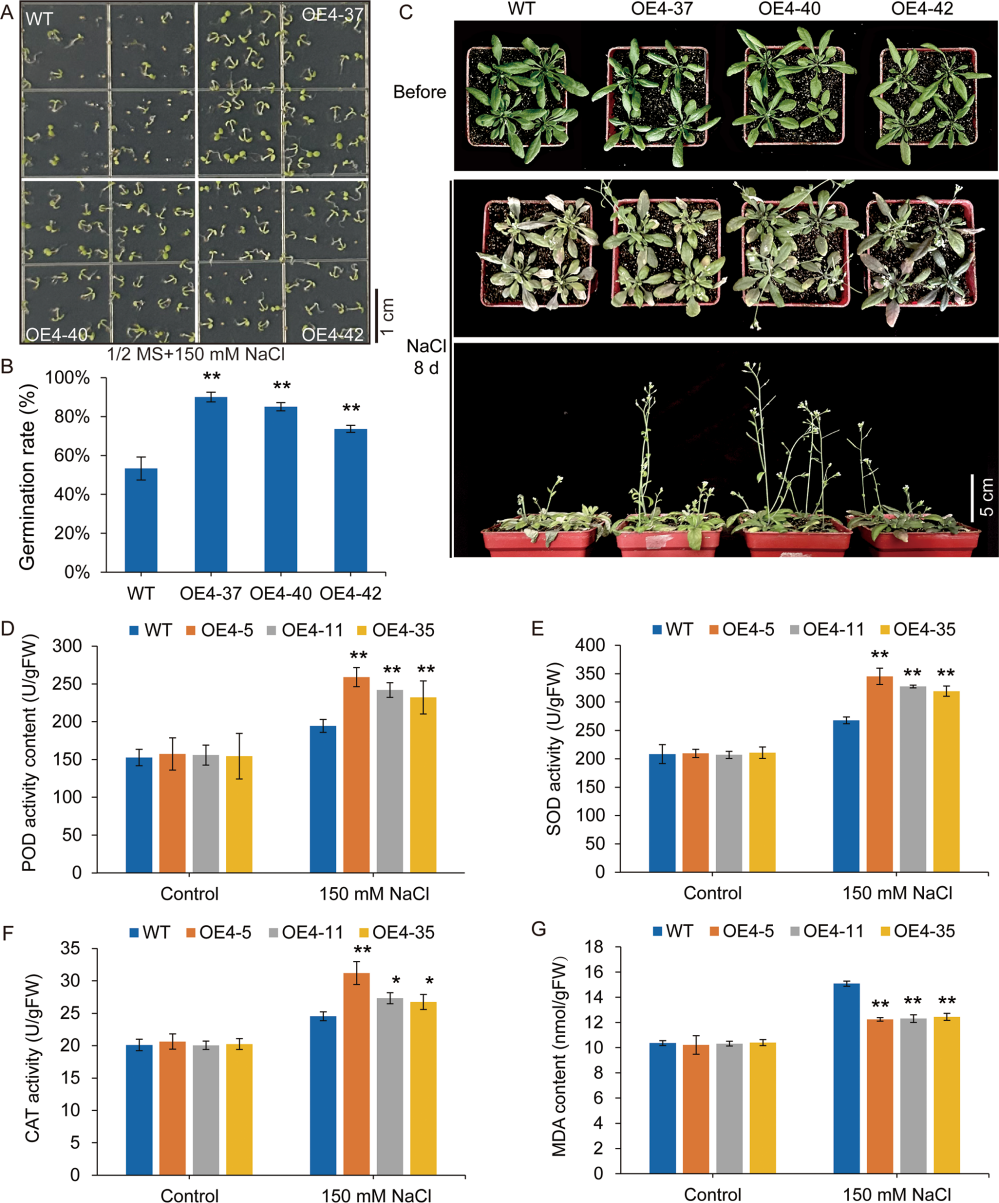
VdSUS4 enhances salt tolerance by strengthening the plant antioxidant system. **(A, B)** Germination rate of WT and transgenic seeds. **(C)** Phenotypic comparison of WT and transgenic lines after treatment with 150 mM NaCl for 8 d. **(D)** POD activity. **(E)** SOD activity. **(F)** CAT activity. **(G)** MDA content. Values are the mean ± standard deviation of three biological replicates. *p*-values < 0.01 are represented by “**” (Student’s t-test). *p*-values < 0.05 are represented by "*".

To explore the potential functions of *VdSUS4* in enhancing plant salt tolerance, we first generated transgenic *Arabidopsis* overexpressing *VdSUS4* (**Supplementary Figure S5**) and assessed their seedling growth under salt stress conditions. Under normal conditions, there were no significant differences in germination and root elongation between wild-type and transgenic seedlings. However, compared with the wild-type, transgenic plants exhibited a nearly 100% increase in root elongation on media containing NaCl (**Figures 7A, B**). And, the fresh weight of transgenic plants demonstrated a 40% increase compared to the wild-type plants (**Figures 7A, C**). Further analysis revealed an exceeding 40% increase in germination rate of transgenic seeds relative to wild-type under 150 mM NaCl treatment (**Figures 8A, B**).

We also performed salt tolerance experiments with wild-type and transgenic plants in soil. The plants were watered with or without 150 mM NaCl solution for 8 days, clearly difference was observed in the leaves and boltings of wild-type and transgenic plants. Both plant groups completed flowering under salt stress, but wild-type plants displayed significantly chlorosis leaves, shorter stems and fewer pods compared to the transgenic lines (**Figure 8C**). All these results indicated that overexpression of *VdSUS4* enhanced salt stress tolerance in Arabidopsis.

Plants adapt to saline-alkaline environments through specific physiological and biochemical regulatory mechanisms. The level of plants salt tolerance can be evaluated by measuring key physiological and biochemical parameters. Our experimental results indicated that, under normal growth conditions, the activities of peroxidase (POD), superoxide dismutase (SOD), and catalase (CAT) showed no significant differences between wild-type and transgenic lines. However, under salt stress conditions, the activities of POD, SOD, and CAT increased in both wild-type and transgenic plants, with significantly higher enzyme activities observed in the transgenic lines. This suggested that plants activated their antioxidant defense systems to mitigate salt-induced oxidative damage, and the transgenic lines possessed a stronger capacity to scavenge reactive oxygen species (ROS), thereby reducing the harmful effects of superoxide radicals and enhancing salt tolerance (**Figures 8D–F**).

Lipid hydroperoxidation serves as an effective indicator of cellular oxidative damage (Yoshimura et al., 2004). Oxidative stress-induced alterations in lipid hydroperoxide accumulation kinetics were assessed through quantitative determination of malondialdehyde (MDA) in foliar disk samples. Upon NaCl stress, malondialdehyde (MDA) concentration markedly increased in wild-type plants, whereas the transgenic lines exhibited only a marginal increase (**Figure 8G**). These findings suggested that *VdSUS4* enhanced the plant’s antioxidant defense system, reduced ROS accumulation, and helped maintain cellular homeostasis, thereby mitigating salt stress-induced damage.

## 4 Discussion

As one of the most common berries, blueberry was domesticated during the 20th century (Miller et al., 2019). Due to its flavor and health-promoting properties, blueberries are now cultivated and consumed worldwide. However, blueberry cultivation still faces various challenges, including the demand for varieties with higher yield and better fruit quality. Moreover, compared to other high-value crops, molecular tools for blueberry breeding are relatively limited, making breeding more challenging (Yocca et al., 2023). In this context, the identification of valuable genetic resources becomes a crucial step in promoting genetic improvement through the application of genetic engineering technologies.

Sucrose synthase (SUS) is widely recognized as a key enzyme involved in sucrose metabolism in higher plants and is considered a biochemical marker for crop strength (Xu et al., 2019). Therefore, conducting systematic study on SUS and identifying candidate genes involved in blueberry fruit ripening and response to abiotic stress is of great significance. In our study, seven members of the *VdSUS* gene family were identified from the blueberry genome (**Table 1**). SUS is a member of the conserved GT-4 glycosyltransferase subfamily, belonging to the larger metal-independent GT-B glycosyltransferase superfamily. In *Arabidopsis*, *AtSUS1* possesses a typical sequence structure of SUS. Similarly, the SUS proteins in blueberries form a symmetrical tetramer (**Supplementary Figure S1D**), with the polypeptide chains folding into four distinct domains. This structural arrangement is well-suited for catalyzing and cleaving sucrose and regulating SUS. Additionally, the secondary structure is predominantly composed of α-helices (**Supplementary Figure S1C**).

Differences in the number of *SUS* genes across species are primarily due to gene duplication and chromosomal segmental or whole-genome duplication events, which lead to varying rates of *SUS* gene birth and loss. This process is a major driving force in species evolution, involving the functional innovation of new genes and the evolution of their expression patterns (Agarwal et al., 2017). Chromosome localization and collinearity analysis suggest that the *SUS* gene family in blueberries may primarily undergo segmental duplication (**Figure 2**). Although genes within a gene family evolve through various mechanisms, comprehensive phylogenetic and structural analysis can provide insights into the evolutionary origin and relationships of different isozymes. Based on their phylogeny and molecular structure, plant SUS proteins have been classified into three major groups (Zou et al., 2013). Our phylogenetic analysis of *VdSUS* genes in blueberry, *Arabidopsis*, rice, tomato, corn, wheat, and sugar beet confirmed this classification (**Supplementary Figure S1F**).

As observed in other plants, such as *Arabidopsis* (Bieniawska et al., 2007) and rice (Hiros et al., 2008), closely related *VdSUS* members generally exhibit similar motifs and exon/intron structures (**Figures 3A, B**), indicating that different SUS proteins may function in a similar manner. However, we also observed differences in genes structure between *VdSUS5* and *VdSUS6*, despite their close proximity in the phylogenetic tree (**Figure 3B**), suggesting that structural divergence among gene family members is caused by mechanisms such as exon/intron loss or gain, insertion/deletion and exonization. Therefore, the analysis of exon/intron structure is crucial for revealing the evolutionary footprint of gene families (Xu et al., 2012). Among the identified 10 motifs, motif 1, 2, 4, 5 and 9 are present in all VdSUS proteins (**Figure 3C**), indicating their high conservation. These motifs constitute the conserved SUS domains essential for the specificity of SUS functions. *Cis*-regulatory elements (CREs) are closely related to gene function and play a crucial role in the transduction of biological signals. In this study, we found that the promoter region of *VdSUS* contains numerous CREs related to hormone regulation, abiotic stress and growth and development (**Figure 4B**), such as response elements related to AAGAA-motif (auxin response), ARE (anaerobic stress-related) and MYC (salt stress-related) (**Figure 4C**), indicating a close correlation between *VdSUS* and the potential regulatory effects on growth and development under different environmental changes.

Functional diversity resulting from gene duplication leads to changes in protein properties and differential expression. This is one of the major evolutionary driving forces for plants to adapt to new environments (Flagel and Wendel, 2009). The expression levels of *VdSUS1* and *VdSUS3* were higher in flowers (YFI and MFI) than other *VdSUS* genes, and both expression levels in MFI were higher than those in YFI (**Figure 5B**), suggesting their potential role in providing energy during the maturation process of flowers. Similarly, *VvSUS4* in grapes is also highly expressed in flowers (Zhu et al., 2017). In fruits, *VdSUS3* shows more expression in MF than those in LGF, while the expression of *VdSUS1* decreases accompanied by the fruit ripening (**Figure 5B**). This phenomenon had been observed in apples as well, where SUS transcription levels were higher in the early stages of fruit development, but decrease as the fruit continues to grow (Li et al., 2012). In the stem, the expression level of *VdSUS1* was significantly higher than that of other members, and its expression in YS was higher than in MS (**Figure 5B**).

In sweet potatoes, *IbSUS3*, *IbSUS4* and *IbSUS8* were highly expressed in the stem (Jiang et al., 2023). These *SUS* gene families were likely involved in carbohydrate transport and assimilation. Additionally, in transgenic poplars, enhanced cellulose deposition leaded to thicker secondary cell walls in the xylem, thereby increasing wood density (Coleman et al., 2009). Sucrose, as the main form of transport for photosynthetic assimilates, was produced in mature leaves, which served as source organs for sucrose synthesis and acted as centers for sucrose export. On the other hand, young leaves, flowers and fruits acted as sink organs, receiving sucrose for development or storing sucrose (Nebauer et al., 2011). In tobacco, *SUS2* and *SUS3* were highly expressed in leaves (Wang et al., 2015). The expression of *VdSUS1* reached peak in young leaves and decreased with leaf development, reaching its lowest level in mature leaves (**Figure 5B**). Meanwhile, *BjSUS5*, *6*, and *7* are significantly overexpressed in young leaves (Koramutla et al., 2019). Similarly, we observed that except for *VdSUS2*, the expression levels of other members in young leaves were higher than those in mature leaves, indicating their unique roles in leaf development. Moreover, in roots, *VdSUS3* exhibits the highest expression level among different *VdSUS* genes, indicating its important role in the development of roots (**Figure 5B**).

An increasing body of research had indicated that the *SUS* gene families were associated with plant responses to environmental stress. Under abiotic stresses such as cold, drought, salinity, and hypoxia, sucrose biosynthesis can protect cell membrane integrity, stabilize proteins, and accelerate metabolism (Strand et al., 2003). During plant recovery from abiotic stress, sucrose serves as an energy source to promote metabolic activity (Strand et al., 2003). Soluble sugars regulate various functions, acting as fuel for growth and development, precursors in metabolism, short- and long-distance signaling molecules, and as components of osmotic protection and reactive oxygen species scavenging systems under stress conditions (Hennion et al., 2019; Salmon et al., 2020). The expression of *AtSUS1* could be induced by cold or drought treatments, and *AtSUS3* could serve as a molecular marker for dehydration (Baud et al., 2004). *HbSUS5* responded to low-temperature and drought stress (Xiao et al., 2014), while *HvSUS1* and *HvSUS3* also responded to low-temperature and drought stress (Barrero-Sicilia et al., 2011). *VvSUS5* was induced by high temperature, cold, salt, darkness and drought conditions (Zhu et al., 2017). *ItbSUS2*, *ItbSUS5* and *ItbSUS6* responded to salt stress (Jiang et al., 2023). In our study, after drought treatment, *VdSUS2*, *VdSUS3, VdSUS4* and *VdSUS5* were highly expressed with increasing treatment time, while *VdSUS1, VdSUS6* and *VdSUS7* gradually decreased (**Figure 6B**). After salt treatment, the expression levels of *VdSUS1*-*VdSUS5* enhanced with increasing treatment time (**Figure 6D**). Under low-temperature treatment, except for *VdSUS5* was induced upregulation, all other genes were significantly downregulated (**Figure 6F**). The expression levels of *SUS* genes might be due to increased demand for glycolysis under abiotic stress conditions (Kleines et al., 1999). The observed downregulation of some *VdSUS* genes in blueberry fruits after sucrose application may be attributed to high concentrations of sucrose acting through energy sensors, such as *SnRK1* kinase, to suppress *SUS* gene expression. This suppression likely serves to prevent further sucrose breakdown, thereby avoiding carbon metabolic imbalance.

In our study, we observed that transgenic *Arabidopsis* overexpressing *VdSUS4* did not exhibit growth and developmental defects, while it enhanced plant tolerance to salt stress (**Figure 7**). The overexpression of *VdSUS4* enhanced salt stress tolerance in *Arabidopsis*, indicating that *VdSUS4* was a valuable candidate gene for improving plant tolerance to abiotic stress through genetic engineering. However, the molecular mechanisms behind this phenomenon are not yet clear, posing an interesting question for future research. In conclusion, our study provides important insights into the *SUS* gene family of blueberry, validates their functions, and lays a theoretical foundation for further functional studies of *SUS* genes in blueberry. This research holds potential applications in future genetic engineering projects.

## 5 Conclusion

In conclusion, our study identified seven *VdSUS* genes within the blueberry genome. Analyses were conducted on their physicochemical properties, phylogenetic relationships, conserved motifs, gene structure, cis-acting elements in promoters and expression patterns to elucidate their potential functions in blueberries. Functional studies in transgenic *Arabidopsis* suggested that *VdSUS* genes may play a role in response to salt stress. Our research findings will provide valuable insights into the response of *VdSUS* genes to various abiotic stresses in blueberry.

## Data Availability

The original contributions presented in the study are included in the article/**supplementary material**. Further inquiries can be directed to the corresponding author.

## Supplementary material

Supplementary Figure 1 Phylogenetic tree of the *SUS* genes family in blueberry and *Arabidopsis.*

Supplementary Figure 2 Characteristics and evolutionary relationships of VdSUS proteins. **(A)** Multiple sequence alignment of VdSUS family proteins. **(B)** Visualization of the Sucrose_synthesis and Glyco_trans_1_4 domains of VdSUS proteins. **(C)** Statistic analysis of the secondary structure of VdSUS amino acid sequences. **(D)** Front view of the overall tertiary structure of VdSUS amino acid sequences. **(E)** Peptide chain structure of VdSUS amino acids.

Supplementary Figure 3 Three-dimensional structures of VdSUS proteins.

Supplementary Figure 4 Expression levels of *VdSUS* genes family after treated with sucrose. Values are the average ± standard deviation of three biological replicates. The transcription levels of *VdSUSs* at 0 h were set as “1”. *P*-values < 0.05 is denoted by “*” respectively (Student’s t-test).

Supplementary Figure 5 Identification of *VdSUS4* transgenic *Arabidopsis*. **(A)** Construct used for *Arabidopsis* transformation. **(B)** PCR verification of different transgenic *Arabidopsis* lines. M: marker, P: *VdSUS4* plasmid, WT: Wild-type. **(C)** RT-PCR confirmed the expression of *VdSUS4* in different transgenic lines. **(D)** qRT-PCR analysed the expression of *VdSUS4* in different transgenic lines.

## References

[B1] Agarwal P. K. Gupta K. Lopato S. Agarwal P. (2017). Dehydration responsive element binding transcription factors and their applications for the engineering of stress tolerance. J. Exp. Bot. 68, 2135–2148. doi: 10.1093/jxb/erx118 28419345

[B2] Albrecht G. Mustroph A. (2003). Localization of sucrose synthase in wheat roots: increased in *situ* activity of sucrose synthase correlates with cell wall thickening by cellulose deposition under hypoxia. Planta 217, 252–260. doi: 10.1007/s00425-003-0995-6 12783333

[B3] An X. M. Chen Z. Wang J. C. Ye M. X. Ji L. X. Wang J.. (2014). Identification and characterization of the Populus sucrose synthase gene family. Gene 539, 58–67. doi: 10.1016/j.gene.2014.01.062 24508272

[B4] Baier M. C. Keck M. Gödde V. Niehaus K. Küster H. Hohnjec N. (2010). Knockdown of the symbiotic sucrose synthase *MtSucS1* affects arbuscule maturation and maintenance in mycorrhizal roots of Medicago truncatula. Plant Physiol. 152, 1000–1014. doi: 10.1104/pp.109.149898 20007443 PMC2815868

[B5] Baroja-Fernández E. Muñoz F. J. Montero M. Etxeberria E. Sesma M. T. Ovecka M.. (2009). Enhancing sucrose synthase activity in transgenic potato (*Solanum tuberosum L.*). tubers results in increased levels of starch., ADPglucose and UDPglucose and total yield. Plant Cell Physiol. 50, 1651–1662. doi: 10.1093/pcp/pcp108 19608713

[B6] Barratt D. H. Barber L. Kruger N. J. Smith A. M. Wang T. L. Martin C. (2001). Multiple, distinct isoforms of sucrose synthase in pea. Plant Physiol. 127, 655–664. doi: 10.1104/pp.127.2.655 11598239 PMC125100

[B7] Barrero-Sicilia C. Hernando-Amado S. González-Melendi P. Carbonero P. (2011). Structure, expression profile and subcellular localisation of four different sucrose synthase genes from barley. Planta 234, 391–403. doi: 10.1007/s00425-011-1408-x 21505865

[B8] Baud S. Vaultier M. N. Rochat C. (2004). Structure and expression profile of the sucrose synthase multigene family in *Arabidopsis* . J. Exp. Bot. 55, 397–409. doi: 10.1093/jxb/erh047 14739263

[B9] Bieniawska Z. Paul Barratt D. H. Garlick A. P. Thole V. Kruger N. J. Martin C.. (2007). Analysis of the sucrose synthase gene family in *Arabidopsis* . Plant J. 49, 810–828. doi: 10.1111/j.1365-313X.2006.03011.x 17257168

[B10] Chen C. Chen H. Zhang Y. Thomas H. R. Frank M. H. He Y.. (2020). TBtools: An integrative toolkit developed for interactive analyses of big biological data. Mol. Plant 13, 1194–1202. doi: 10.1016/j.molp.2020.06.009 32585190

[B11] Chen A. He S. Li F. Li Z. Ding M. Liu Q.. (2012). Analyses of the sucrose synthase gene family in cotton: structure, phylogeny and expression patterns. BMC Plant Biol. 12, 85. doi: 10.1186/1471-2229-12-85 22694895 PMC3505178

[B12] Chourey P. S. Taliercio E. W. Carlson S. J. Ruan Y. L. (1998). Genetic evidence that the two isozymes of sucrose synthase present in developing maize endosperm are critical, one for cell wall integrity and the other for starch biosynthesis. Mol. Gen. Genet. 259, 88–96. doi: 10.1007/s004380050792 9738884

[B13] Ciereszko I. Johansson H. Kleczkowski L. A. (2001). Sucrose and light regulation of a cold-inducible UDP-glucose pyrophosphorylase gene via a hexokinase-independent and abscisic acid-insensitive pathway in *Arabidopsis* . Biochem. J. 354, 67–72. doi: 10.1042/0264-6021:3540067 11171080 PMC1221629

[B14] Clough S. J. Bent A. F. (1998). Floral dip: a simplified method for Agrobacterium-mediated transformation of *Arabidopsis thaliana* . Plant J. 16, 735–743. doi: 10.1046/j.1365-313x.1998.00343.x 10069079

[B15] Coleman H. D. Yan J. Mansfield S. D. (2009). Sucrose synthase affects carbon partitioning to increase cellulose production and altered cell wall ultrastructure. Proc. Natl. Acad. Sci. U. S. A 106, 13118–13123. doi: 10.1073/pnas.0900188106 19625620 PMC2722352

[B16] Duan Y. Tarafdar A. Chaurasia D. Singh A. Bhargava P. C. Yang J. F.. (2022). Blueberry fruit valorization and valuable constituents: A review. Int. J. Food Microbiol. 381, 109890. doi: 10.1016/j.ijfoodmicro.2022.109890 36095867

[B17] Duan Y. Yang L. Zhu H. Zhou J. Sun H. Gong H. (2021). Structure and expression analysis of sucrose phosphate synthase, sucrose synthase and invertase gene families in *Solanum lycopersicum* . Int. J. Mol. Sci. 22, 4698. doi: 10.3390/ijms22094698 33946733 PMC8124378

[B18] Duncan K. A. Hardin S. C. Huber S. C. (2006). The three maize sucrose synthase isoforms differ in distribution, localization, and phosphorylation. Plant Cell Physiol. 47, 959–971. doi: 10.1093/pcp/pcj068 16760218

[B19] Figueroa C. M. Lunn J. E. (2016). A tale of two sugars: trehalose 6-phosphate and sucrose. Plant Physiol. 172, 7–27. doi: 10.1104/pp.16.00417 27482078 PMC5074632

[B20] Flagel L. E. Wendel J. F. (2009). Gene duplication and evolutionary novelty in plants. New Phytol. 183, 557–564. doi: 10.1111/j.1469-8137.2009.02923.x 19555435

[B21] Gaudin V. Lunness P. A. Fobert P. R. Towers M. Riou-Khamlichi C. Murray J. A.. (2000). The expression of D-cyclin genes defines distinct developmental zones in snapdragon apical meristems and is locally regulated by the Cycloidea gene. Plant Physiol. 122, 1137–1148. doi: 10.1104/pp.122.4.1137 10759509 PMC58948

[B22] Gerber L. Zhang B. Roach M. Rende U. Gorzsás A. Kumar M.. (2014). Deficient sucrose synthase activity in developing wood does not specifically affect cellulose biosynthesis but causes an overall decrease in cell wall polymers. New Phytol. 203, 1220–1230. doi: 10.1111/nph.12888 24920335

[B23] Haigler C. H. Ivanova-Datcheva M. Hogan P. S. Salnikov V. V. Hwang S. Martin K.. (2001). Carbon partitioning to cellulose synthesis. Plant Mol. Biol. 47, 29–51. doi: 10.1023/A:1010615027986 11554477

[B24] Harada T. Satoh S. Yoshioka T. Ishizawa K. (2005). Expression of sucrose synthase genes involved in enhanced elongation of pondweed (*Potamogeton distinctus*). turions under anoxia. Ann. Bot. 96, 683–692. doi: 10.1093/aob/mci220 16033779 PMC4247035

[B25] Hennion N. Durand M. Vriet C. Doidy J. Maurousset L. Lemoine R.. (2019). Sugars en route to the roots. Transport, metabolism and storage within plant roots and towards microorganisms of the rhizosphere. Physiol. Plant 165, 44–57. doi: 10.1111/ppl.12751 29704246

[B26] Higo K. Ugawa Y. Iwamoto M. Korenaga T. (1999). Plant cis-acting regulatory DNA elements (PLACE). database: 1999. Nucleic Acids Res. 27, 297–300. doi: 10.1093/nar/27.1.297 9847208 PMC148163

[B27] Hiros T. Scofield G. N. Terao T. (2008). An expression analysis profile for the entire sucrose synthase gene family in rice. Plant Sci. 174, 534–543. doi: 10.1016/j.plantsci.2008.02.009

[B28] Huang T. Luo X. Fan Z. Yang Y. Wan W. (2021). Genome-wide identification and analysis of the sucrose synthase gene family in cassava (*Manihot esculenta Crantz*). Gene 769, 145191. doi: 10.1016/j.gene.2020.145191 33007377

[B29] Huber S. C. Huber J. L. (1996). Role and regulation of sucrose-phosphate synthase in higher plants. Annu. Rev. Plant Physiol. Plant Mol. Biol. 47, 431–444. doi: 10.1146/annurev.arplant.47.1.431 15012296

[B30] Iraqi D. Tremblay F. M. (2001). Analysis of carbohydrate metabolism enzymes and cellular contents of sugars and proteins during spruce somatic embryogenesis suggests a regulatory role of exogenous sucrose in embryo development. J. Exp. Bot. 52, 2301–2311. doi: 10.1093/jexbot/52.365.2301 11709580

[B31] Islam M. Z. Hu X. M. Jin L. F. Liu Y. Z. Peng S. A. (2014). Genome-wide identification and expression profile analysis of citrus sucrose synthase genes: investigation of possible roles in the regulation of sugar accumulation. PloS One 9, e113623. doi: 10.1371/journal.,pone.0113623 25420091 PMC4242728

[B32] Jia H. Jiu S. Zhang C. Wang C. Tariq P. Liu Z.. (2016). Abscisic acid and sucrose regulate tomato and strawberry fruit ripening through the abscisic acid-stress-ripening transcription factor. Plant Biotechnol. J. 14, 2045–2065. doi: 10.1111/pbi.12563 27005823 PMC5043491

[B33] Jia H. Wang Y. Sun M. Li B. Han Y. Zhao Y.. (2013). Sucrose functions as a signal involved in the regulation of strawberry fruit development and ripening. New Phytol. 198, 453–465. doi: 10.1111/nph.12176 23425297

[B34] Jiang Z. Zhang H. Gao S. Zhai H. He S. Zhao N.. (2023). Genome-Wide identification and expression analysis of the sucrose synthase gene family in sweet potato and its two diploid relatives. Int. J. Mol. Sci. 24, 12493. doi: 10.3390/ijms241512493 37569874 PMC10420203

[B35] Kleines M. Elster R. C. Rodrigo M. J. Blervacq A. S. Salamini F. Bartels D. (1999). Isolation and expression analysis of two stress-responsive sucrose-synthase genes from the resurrection plant Craterostigma plantagineum (Hochst.). Planta 209, 13–24. doi: 10.1007/s004250050602 10467027

[B36] Koramutla M. K. Ram C. Bhatt D. Annamalai M. Bhattacharya R. (2019). Genome-wide identification and expression analysis of sucrose synthase genes in allotetraploid Brassica juncea. Gene 707, 126–135. doi: 10.1016/j.gene.2019.04.059 31026572

[B37] Kumar S. Stecher G. Li M. Knyaz C. Tamura K. (2018). MEGA X: Molecular evolutionary genetics analysis across computing platforms. Mol. Biol. Evol. 35, 1547–1549. doi: 10.1093/molbev/msy096 29722887 PMC5967553

[B38] Li M. Feng F. Cheng L. (2012). Expression patterns of genes involved in sugar metabolism and accumulation during apple fruit development. PloS One 7, e33055. doi: 10.1371/journal.,pone.0033055 22412983 PMC3296772

[B39] Li F. Hao C. Yan L. Wu B. Qin X. Lai J.. (2015). Gene structure, phylogeny and expression profile of the sucrose synthase gene family in cacao (*Theobroma cacao L.*). J. Genet. 94, 461–472. doi: 10.1007/s12041-015-0558-1 26440085

[B40] Li P. Ma H. Xiao N. Zhang Y. Xu T. Xia T. (2023). Overexpression of the *ZmSUS1* gene alters the content and composition of endosperm starch in maize (*Zea mays L.*). Planta 257, 97. doi: 10.1007/s00425-023-04133-z 37052727

[B41] Li M. Wang S. Liu Y. Zhang Y. Ren M. Liu L.. (2019). Overexpression of *PsnSuSy1*, 2 genes enhances secondary cell wall thickening, vegetative growth, and mechanical strength in transgenic tobacco. Plant Mol. Biol. 100, 215–230. doi: 10.1007/s11103-023-01353-5 31053988

[B42] Liu L. Zheng J. (2022). Identification and expression analysis of the sucrose synthase gene family in pomegranate (*Punica granatum L.*). PeerJ 10, e12814. doi: 10.7717/peerj.12814 35047243 PMC8757371

[B43] Lunn J. E. MacRae E. (2003). New complexities in the synthesis of sucrose. Curr. Opin. Plant Biol. 6, 208–214. doi: 10.1016/s1369-5266(03)00033-5 12753969

[B44] Miller K. Feucht W. Schmid M. (2019). Bioactive compounds of strawberry and blueberry and their potential health effects based on human intervention studies: A brief overview. Nutrients 11, 1510. doi: 10.3390/nu11071510 31269727 PMC6683271

[B45] Nagaraj V. J. Riedl R. Boller T. Wiemken A. Meyer A. D. (2001). Light and sugar regulation of the barley sucrose: fructan-6-fructosyltransferase promoter. J. Plant Physiol. 158, 1601–1607. doi: 10.1078/0176-1617-00592

[B46] Nebauer S. G. Renau-Morata B. Guardiola J. L. Molina R. V. (2011). Photosynthesis down-regulation precedes carbohydrate accumulation under sink limitation in Citrus. Tree Physiol. 31, 169–177. doi: 10.1093/treephys/tpq103 21367744

[B47] Rook F. Corke F. Card R. Munz G. Smith C. Bevan M. W. (2001). Impaired sucrose-induction mutants reveal the modulation of sugar-induced starch biosynthetic gene expression by abscisic acid signalling. Plant J. 26, 421–433. doi: 10.1046/j.1365-313x 11439129

[B48] Salmon Y. Lintunen A. Dayet A. Chan T. Dewar R. Vesala T.. (2020). Leaf carbon and water status control stomatal and nonstomatal limitations of photosynthesis in trees. New Phytol. 226, 690–703. doi: 10.1111/nph.16436 31955422

[B49] Schmittgen T. D. Livak K. J. (2008). Analyzing real-time PCR data by the comparative C(T). method. Nat. Protoc. 3, 1101–1108. doi: 10.1038/nprot.2008.73 18546601

[B50] Shen L. Zhang L. Yin C. Xu X. Liu Y. Shen K.. (2023). The wheat sucrose synthase gene *TaSus1* is a determinant of grain number per spike. Crop J. 12, 295–300. doi: 10.1016/j.cj.2023.11.007

[B51] Stein O. Granot D. (2019). An overview of sucrose synthases in plants. Front. Plant Sci. 10. doi: 10.3389/fpls.2019.00095 PMC6375876 30800137

[B52] Strand Å. Foyer C. H. Gustafsson P. GardestrÖM P. Hurry V. (2003). Altering flux through the sucrose biosynthesis pathway in transgenic Arabidopsis thaliana modifies photosynthetic acclimation at low temperatures and the development of freezing tolerance. Plant Cell Environ. 26, 523–535. doi: 10.1046/j.1365-3040.2003.00983.x

[B53] Tong X. Wang Z. Ma B. Zhang C. Zhu L. Ma F.. (2018). Structure and expression analysis of the sucrose synthase gene family in apple. J. Integr. Agr. 17, 847–856. doi: 10.1016/S2095-3119(17)61755-6

[B54] Wang Z. Wei P. Wu M. Xu Y. Li F. Luo Z.. (2015). Analysis of the sucrose synthase gene family in tobacco: structure, phylogeny, and expression patterns. Planta 242, 153–166. doi: 10.1007/s00425-015-2297-1 25893870 PMC4471321

[B55] Wang H. Sui X. Guo J. Wang Z. Cheng J. Ma S.. (2014). Antisense suppression of cucumber (*Cucumis sativus L.*). *sucrose synthase* 3 (*CsSUS3*). reduces hypoxic stress tolerance. Plant Cell Environ. 37, 795–810. doi: 10.1111/pce.12200 24028217

[B56] Xiao X. Tang C. Fang Y. Yang M. Zhou B. Qi J.. (2014). Structure and expression profile of the sucrose synthase gene family in the rubber tree: indicative of roles in stress response and sucrose utilization in the laticifers. FEBS. J. 281, 291–305. doi: 10.1111/febs.12595 24279382

[B57] Xu S. M. Brill E. Llewellyn D. J. Furbank R. T. Ruan Y. L. (2012). Overexpression of a potato sucrose synthase gene in cotton accelerates leaf expansion, reduces seed abortion, and enhances fiber production. Mol. Plant 5, 430–441. doi: 10.1093/mp/ssr090 22115917

[B58] Xu X. Yang Y. Liu C. Sun Y. Zhang T. Hou M.. (2019). The evolutionary history of the sucrose synthase gene family in higher plants. BMC Plant Biol. 19, 566. doi: 10.1186/s12870-019-2181-4 31852440 PMC6921546

[B59] Yadav U. P. Ivakov A. Feil R. Duan G. Y. Walther D. Giavalisco P.. (2014). The sucrose-trehalose-6-phosphate (Tre6P). nexus: specificity and mechanisms of sucrose signalling by Tre6P. J. Exp. Bot. 65, 1051–1068. doi: 10.1093/jxb/ert457 24420566 PMC3935566

[B60] Yang J. Zhang J. Wang Z. Zhu Q. (2001). Activities of starch hydrolytic enzymes and sucrose-phosphate synthase in the stems of rice subjected to water stress during grain filling. J. Exp. Bot. 52, 2169–2179. doi: 10.1093/jexbot/52.364.2169 11604456

[B61] Yocca A. E. Platts. A. Alger E. Teresi S. Mengist M. F. Benevenuto J.. (2023). Blueberry and cranberry pangenomes as a resource for future genetic studies and breeding efforts. Hortic. Res. 10, 202. doi: 10.1093/hr/uhad202 PMC10673653 38023484

[B62] Yoon J. Cho L. H. Tun W. Jeon J. S. An G. (2021). Sucrose signaling in higher plants. Plant Sci. 302, 110703. doi: 10.1016/j.plantsci.2020.110703 33288016

[B63] Yoshimura K. Miyao K. Gaber A. Takeda T. Shigeoka S. (2004). Enhancement of stress tolerance in transgenic tobacco plants overexpressing Chlamydomonas glutathione peroxidase in chloroplasts or cytosol. Plant J. 37, 21–33. doi: 10.1046/j.1365-313X.2003.01930.x 14675429

[B64] \Zhao H. Gao Z. Wang L. Wang J. Wang S. Fei B.. (2018). Chromosome-level reference genome and alternative splicing atlas of moso bamboo. Gigascience 7, 115. doi: 10.1093/gigascience/giy115 PMC6204424 30202850

[B65] Zheng Y. Anderson S. Zhang Y. Garavito R. M. (2011). The structure of sucrose synthase-1 from *Arabidopsis thaliana* and its functional implications. J. Biol. Chem. 286, 36108–36118. doi: 10.1074/jbc.M111.275974 21865170 PMC3195635

[B66] Zhu X. Wang M. Li X. Jiu S. Wang C. Fang J. (2017). Genome-wide analysis of the sucrose synthase gene family in grape (*Vitis vinifera*). Structure, evolution, and expression profiles. Genes 8, 111. doi: 10.3390/genes8040111 28350372 PMC5406858

[B67] Zou C. Lu C. Shang H. Jing X. Cheng H. Zhang Y.. (2013). Genome-wide analysis of the *Sus* gene family in cotton. J. Integr. Plant Biol. 55, 643–653. doi: 10.1111/jipb.12068 23691964

[B68] Zrenner R. Salanoubat M. Willmitzer L. Sonnewald U. (1995). Evidence of the crucial role of sucrose synthase for sink strength using transgenic potato plants (*Solanum tuberosum L.*). Plant J. 7, 97–107. doi: 10.1046/j.1365-313x.1995.07010097.x 7894514

