# Supplementary figures and images for: The sucrose synthase gene family in blueberry (Vaccinium darrowii): functional insights into the role of VdSUS4 in salt stress tolerance
Source: Front Plant Sci. 2025 Jun 2;16:1581182. doi: 10.3389/fpls.2025.1581182 (PMC12171176; doi:10.3389/fpls.2025.1581182)

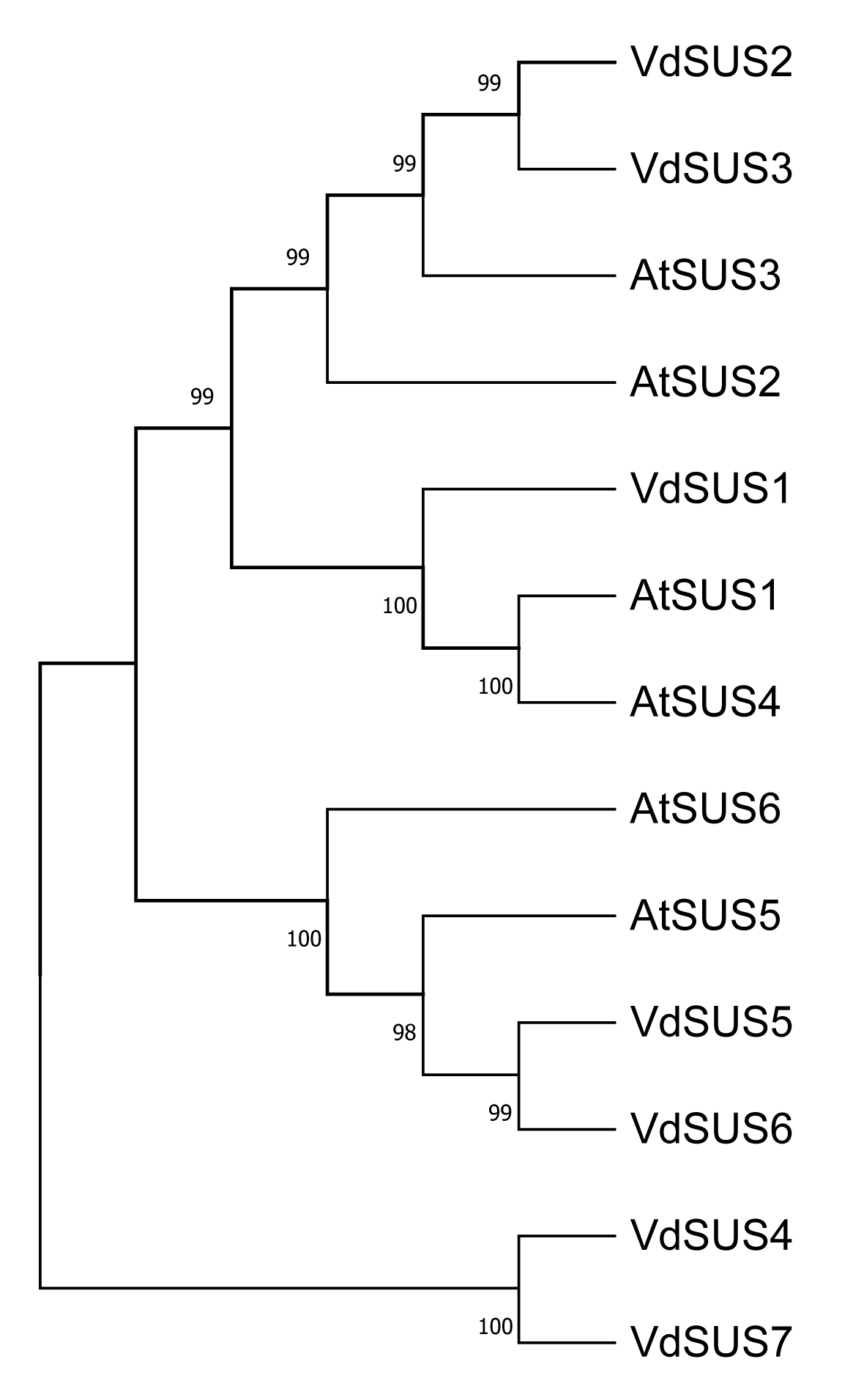

Supplement: Supplementary Figure 1 — Phylogenetic tree of the SUS genes family in blueberry and Arabidopsis. [file Image1.tif]

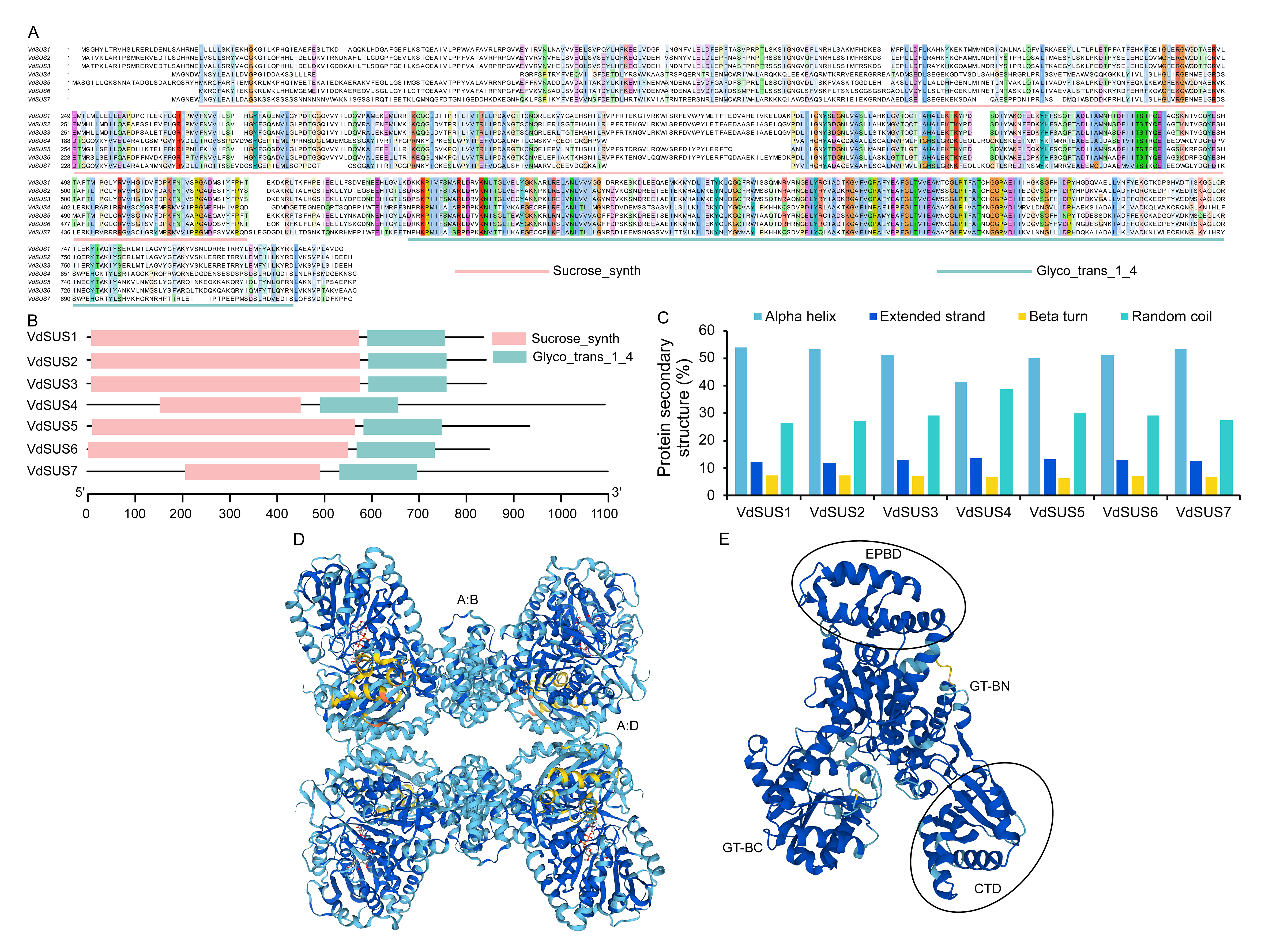

Supplement: Supplementary Figure 2 — Characteristics and evolutionary relationships of VdSUS proteins. (A) Multiple sequence alignment of VdSUS family proteins. (B) Visualization of the Sucrose_synthesis and Glyco_trans_1_4 domains of VdSUS proteins. (C) Statistic analysis of the secondary structure of VdSUS amino acid sequences. (D) Front view of the overall tertiary structure of VdSUS amino acid sequences. (E) Peptide chain structure of VdSUS amino acids. [file Image2.tif]

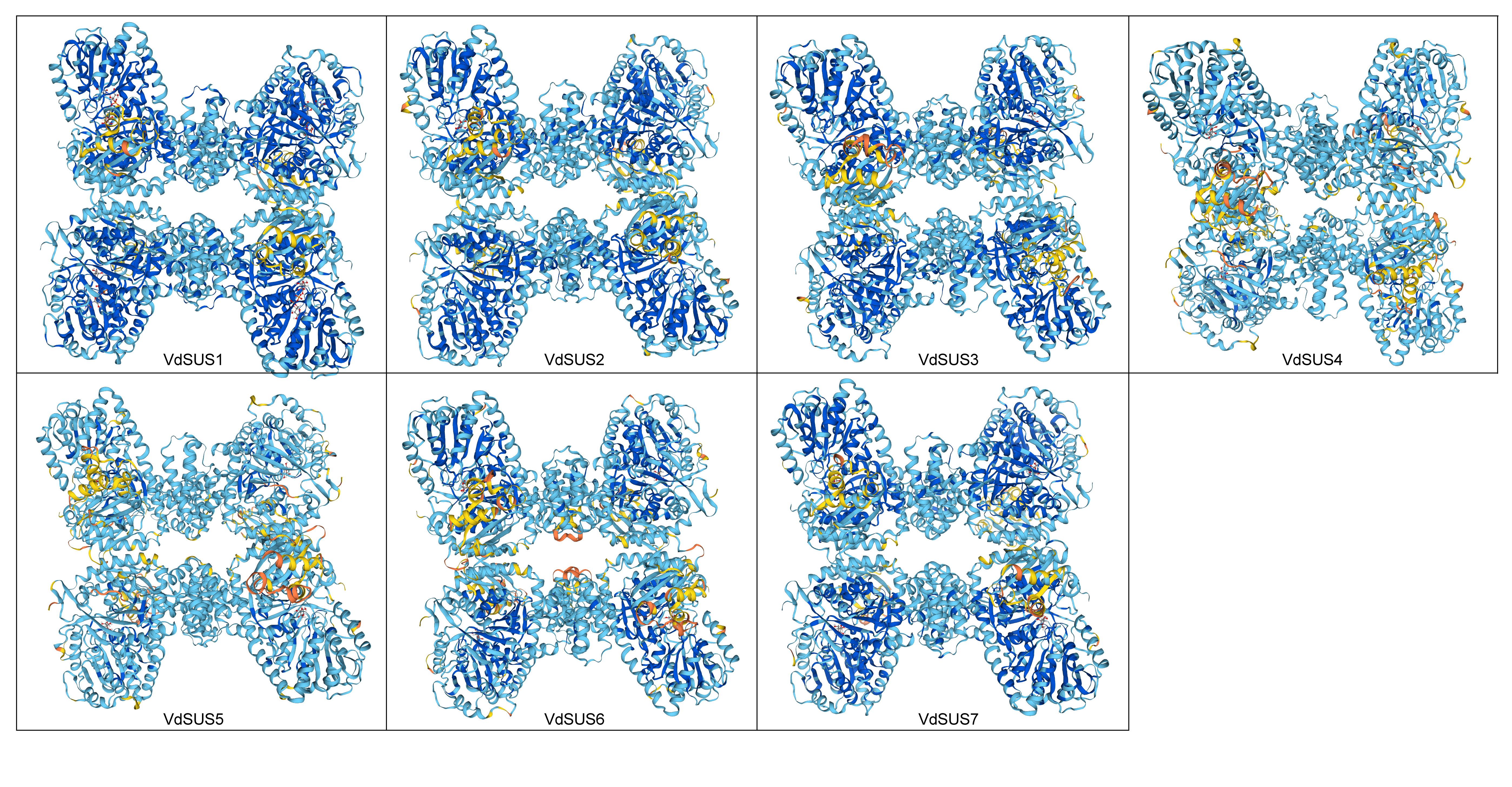

Supplement: Supplementary Figure 3 — Three-dimensional structures of VdSUS proteins. [file Image3.tif]

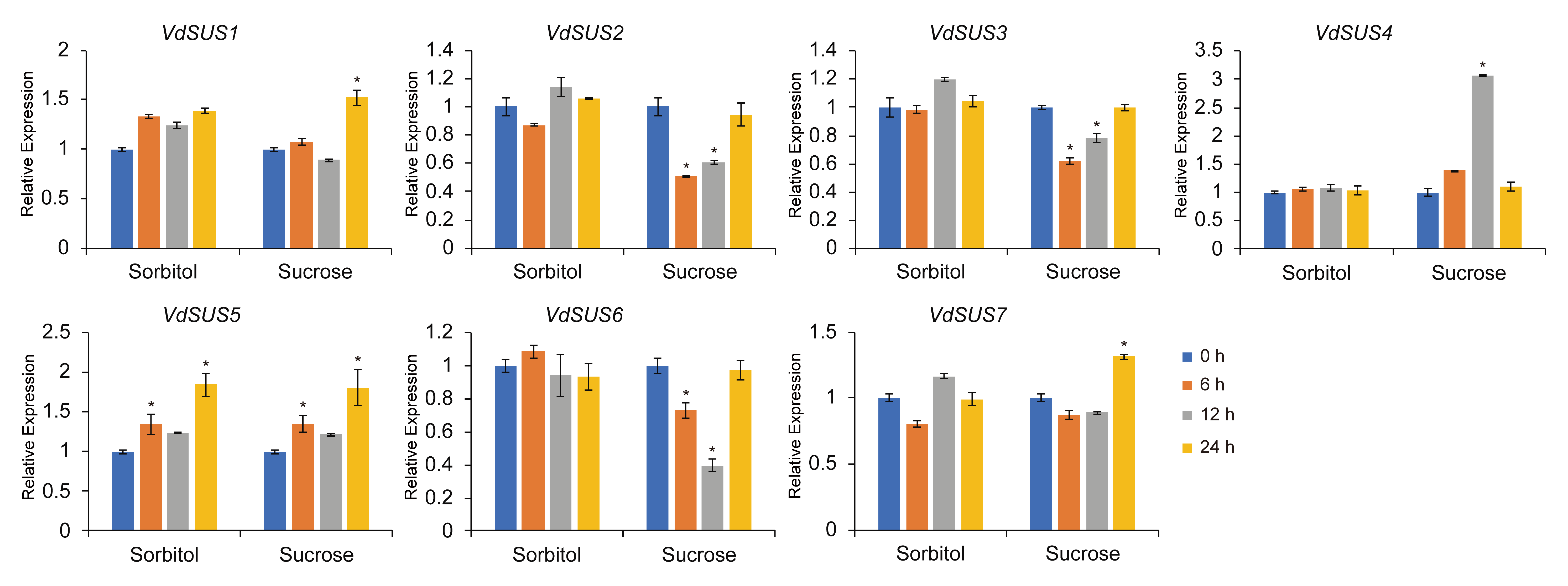

Supplement: Supplementary Figure 4 — Expression levels of VdSUS genes family after treated with sucrose. Values are the average ± standard deviation of three biological replicates. The transcription levels of VdSUSs at 0 h were set as “1”. P-values < 0.05 is denoted by “*” respectively (Student’s t-test). [file Image4.tif]

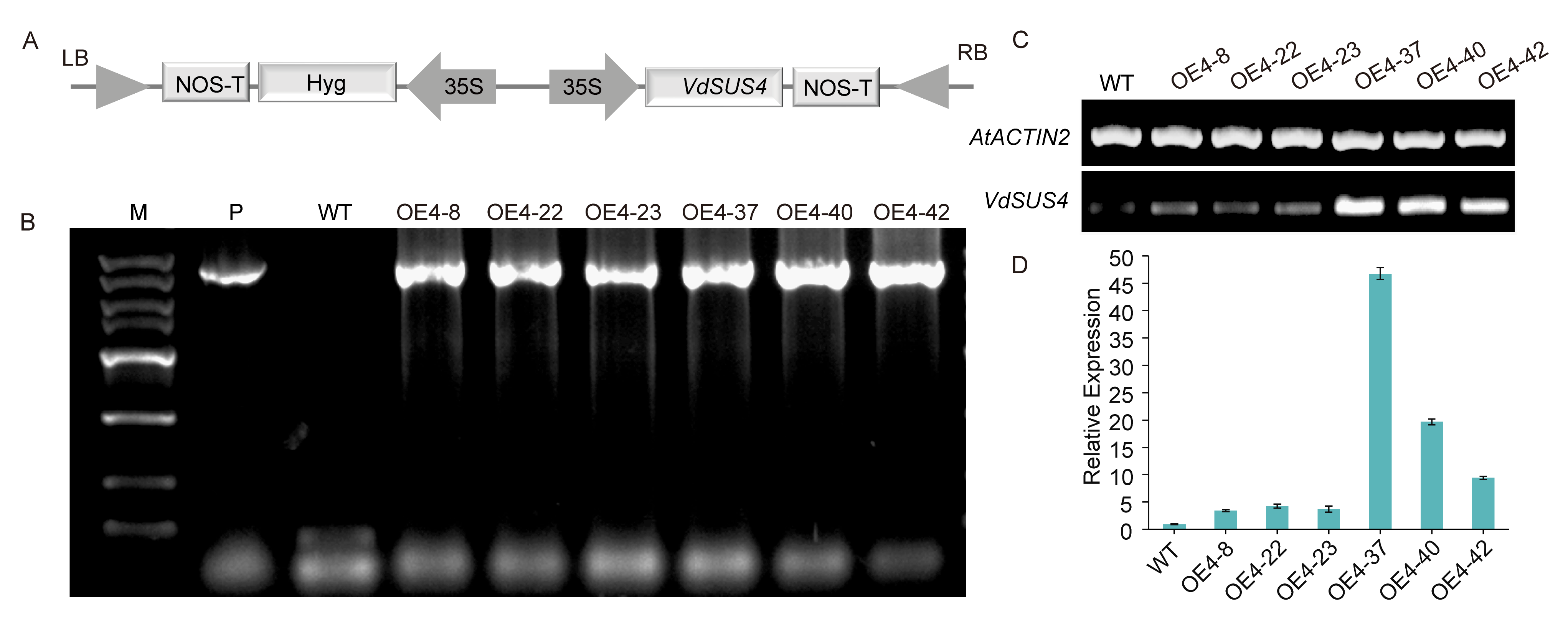

Supplement: Supplementary Figure 5 — Identification of VdSUS4 transgenic Arabidopsis. (A) Construct used for Arabidopsis transformation. (B) PCR verification of different transgenic Arabidopsis lines. M: marker, P: VdSUS4 plasmid, WT: Wild-type. (C) RT-PCR confirmed the expression of VdSUS4 in different transgenic lines. (D) qRT-PCR analysed the expression of VdSUS4 in different transgenic lines. [file Image5.tif]
